# Efficacy of a Yeast Cell Wall Extract to Mitigate the Effect of Naturally Co-Occurring Mycotoxins Contaminating Feed Ingredients Fed to Young Pigs: Impact on Gut Health, Microbiome, and Growth

**DOI:** 10.3390/toxins11110633

**Published:** 2019-10-31

**Authors:** Sung Woo Kim, Débora Muratori Holanda, Xin Gao, Inkyung Park, Alexandros Yiannikouris

**Affiliations:** 1 Department of Animal Science, North Carolina State University, Raleigh, NC 27695, USA; dmurato@ncsu.edu (D.M.H.); ipark2@ncsu.edu (I.P.); 2 Alltech Inc, Center for Animal Nutrigenomics and Applied Animal Nutrition, 3031 Catnip Hill Road, Nicholasville, KY 40356, USA; ayiannikouris@alltech.com

**Keywords:** mycotoxin, prevention, reduction strategies

## Abstract

Mycotoxins are produced by fungi and are potentially toxic to pigs. Yeast cell wall extract (YCWE) is known to adsorb mycotoxins and improve gut health in pigs. One hundred and twenty growing (56 kg; experiment 1) and 48 nursery piglets (6 kg; experiment 2) were assigned to four dietary treatments in a 2 × 2 factorial design for 35 and 48 days, respectively. Factors were mycotoxins (no addition versus experiment 1: 180 μg/kg aflatoxins and 14 mg/kg fumonisins; or experiment 2: 180 μg/kg aflatoxins and 9 mg/kg fumonisins, and 1 mg/kg deoxynivalenol) and YCWE (0% versus 0.2%). Growth performance, blood, gut health and microbiome, and apparent ileal digestibility (AID) data were evaluated. In experiment 1, mycotoxins reduced ADG and G:F, and duodenal IgG, whereas in jejunum, YCWE increased IgG and reduced villus width. In experiment 2, mycotoxins reduced BW, ADG, and ADFI. Mycotoxins reduced ADG, which was recovered by YCWE. Mycotoxins reduced the AID of nutrients evaluated and increased protein carbonyl, whereas mycotoxins and YCWE increased the AID of the nutrients and reduced protein carbonyl. Mycotoxins reduced villus height, proportion of Ki-67-positive cells, and increased IgA and the proportion of bacteria with mycotoxin-degrading ability, whereas YCWE tended to increase villus height and reduced IgA and the proportion of pathogenic bacteria in jejunum. The YCWE effects were more evident in promoting gut health and growth in nursery pigs, which showed higher susceptibility to mycotoxin effects.

## 1. Introduction

Mycotoxins are toxic secondary metabolites produced by certain species of fungi growing on cereal grains and feedstuffs [1]. Cereal grains such as corn, sorghum, and wheat have been used in the United States as the main feedstuffs in swine production. However, these feedstuffs are frequently contaminated with several types of co-occurring mycotoxins and contribute significantly to the overall contamination in compound feeds and potential impact on animal performance and health. Amongst hundreds of mycotoxins potentially contaminating feedstuffs, the response in pigs to the presence of aflatoxin B1 (AFB1), deoxynivalenol (DON), and fumonisin B1 (FB1) has been well documented [1]. Mycotoxicosis can impact gut health, alter immune function, and susceptibility of the animal to other contaminants or pathogens causing organ damage in pigs. The harmful effects of mycotoxin eventually lead to reduced growth performance of pigs [1,2,3,4].

Although feeding pigs without mycotoxins is the ultimate approach to counteract their impact, mycotoxin contamination in feeds is unavoidable [5]. In the United States, the Food and Drug Administration (FDA) limits aflatoxins (AF) concentration for immature pigs at 20 μg/kg of feed. For DON and fumonisins (FUM), there is no upper limit, but the FDA has advisory levels of 1 mg/kg of feed and 2 mg/kg of feed for DON and FUM, respectively. Therefore, various strategies to reduce mycotoxicosis have been investigated in pig production. Adsorbents have been used to mitigate mycotoxicosis by directly decreasing the mycotoxin bioavailability, and consequently, by indirectly reducing the inflammatory response, improving intestinal health, and by preventing oxidative stress [1,2,3,4]. Organic adsorbents, such as yeast cell wall and algae-based carbohydrates have shown that their ß-D-glucans composition and tridimensional network were able to chemically adsorb mycotoxins in vitro [6,7,8,9], reduce the absorption of mycotoxins in the small intestine [10], decrease the accumulation of mycotoxins in specific organs, and increase their clearance [11], thus protecting the vital organs against mycotoxin exposure. Thus, it is hypothesized that yeast cell wall extract (YCWE) supplementation might mitigate the adverse effects of pig diets naturally contaminated with mycotoxins.

The objective of this study was to determine the effects of YCWE (Mycosorb™ A+, Alltech, KY, USA) derived from the cell wall of *Saccharomyces cerevisiae* and a heterotrophically grown microalgae on the growth performances and gut health variables, such as gut integrity and permeability, oxidative stress, immune response, and microbiome in pigs fed diets with naturally contaminated mycotoxins (AFB1, DON, and FB1).

## 2. Results

### 2.1. Experiment 1

#### 2.1.1. Growth Performance

There were no effects of mycotoxins, YCWE, or interaction in body weights of pigs during the first 7 days (Table 1). From day 7 to 14, feeding diets with mycotoxins tended to reduce (*p* = 0.099) body weight, but this trend did not continue during the later periods. From day 21 to 35, there were no effects of mycotoxins, YCWE, or interaction in body weights of pigs.

During the first 7 days, feeding diets with mycotoxins reduced (*p* < 0.05) average daily gain (ADG) and feeding diets with YCWE tended to reduce (*p* = 0.088) ADG. However, there were no effects of mycotoxins, YCWE, or interaction in ADG of pigs after the first 7 days. There were no effects of mycotoxins, YCWE, or interaction in average daily feed intake (ADFI) of pigs until day 28, whereas feeding diets with YCWE tended to further reduce (*p* = 0.073) the ADFI of pigs during days 28 to 35. Feeding diets with mycotoxins reduced (*p* < 0.05) the gain to feed ratio (G:F) of pigs during the first 7 days, whereas it tended to increase (*p* = 0.098) G:F during days 14 to 21. During the entire 35-day period, there were no effects of mycotoxins, YCWE, or interaction in ADG, ADFI, and G:F of pigs.

#### 2.1.2. Hematological Measurements

Feeding diets with mycotoxins reduced (*p* < 0.05) neutrophils blood levels on day 28 (Table 2). Feeding diets with YCWE did not influence cell counts in blood on day 28. There was a tendency (*p* = 0.099) for an interaction between feeding diets with mycotoxins and with YCWE in the lymphocyte count in blood, indicating that YCWE tended to increase (*p* < 0.10) lymphocyte count in pigs fed diets without mycotoxins, whereas this tendency disappeared when mycotoxins were introduced. There were no effects of mycotoxins, YCWE, or interaction in red or white blood cells, hemoglobin, hematocrit, mean corpuscular volume, mean corpuscular hemoglobin, mean corpuscular hemoglobin concentration, platelet, monocytes, eosinophils, or basophils of pigs.

#### 2.1.3. Serum Biochemical Measurements

Feeding diets with mycotoxins increased (*p* < 0.05) serum albumin concentration of pigs, and there was a tendency (*p* = 0.074) for an interaction between feeding diets with mycotoxins and with YCWE for albumin on day 28 (Table 3). Feeding diets with YCWE reduced serum albumin in pigs fed diets without mycotoxins, whereas feeding diets with YCWE increased serum albumin in pigs fed diets with mycotoxins. There were no effects of mycotoxins, YCWE, or interaction in globulin, albumin-to-globulin ratio, total protein, or most hepatic enzymes evaluated (aspartate aminotransferase, alanine aminotransferase, and creatine phosphokinase) in serum of pigs. Feeding diets with mycotoxins tended to increase (*p* = 0.098) alkaline phosphatase, tended to reduce (*p* = 0.088) blood urea nitrogen (BUN), and tended to increase (*p* = 0.051) glucose in serum of pigs. Feeding diets with mycotoxins decreased (*p* < 0.05) cholesterol level in serum of pigs. There were no effects of mycotoxins, YCWE, or interaction in creatinine or BUN-to-creatinine ratio in serum of pigs. Feeding diets with YCWE tended to increase chloride (*p* = 0.081) and sodium (*p* = 0.064) in serum. Feeding diets with mycotoxins tended to decrease (*p* = 0.064) potassium and tended to increase (*p* = 0.053) sodium-to-potassium ratio in serum. There were no effects of mycotoxins, YCWE, or interaction in calcium levels of pigs.

#### 2.1.4. Immunological and Oxidative Stress Measurements

No effects of mycotoxins, YCWE, or interaction were found in the concentrations of tumor necrosis factor-alpha (TNF-α) in duodenal and jejunal mucosa as well as in serum of pigs (Table 4). Feeding diets with mycotoxins increased (*p* < 0.05) duodenal immunoglobulin G (IgG). Feeding diets with YCWE reduced (*p* < 0.05) serum 8-hydroxy-2′-deoxyguanosine (8-OHdG) of pigs. For instance, in pigs fed diets with mycotoxins, there was a reduction of the levels of serum 8-OHdG down to 0.26 ng/mL when YCWE was included, whereas when no YCWE was added, there was increased oxidation with up to 1.66 ng/mL of 8-OHdG. There was an interaction (*p* < 0.05) between mycotoxins and YCWE, where a reduced (*p* < 0.05) jejunal IgG concentration was observed in pigs fed diets with mycotoxins with the addition of YCWE, whereas feeding with YCWE increased (*p* < 0.05) jejunal IgG levels in animals not challenged with mycotoxins. A tendency (*p* = 0.096) for an interaction was observed for serum IgG, indicating that YCWE increased (*p* < 0.05) serum IgG concentration in pigs, whereas this effect disappeared when mycotoxins were introduced. No effects of mycotoxins, YCWE, or interaction were observed on oxidative stress, concentrations of malondialdehydes (MDA) in duodenal and jejunal mucosa or in serum of pigs.

#### 2.1.5. Histomorphometry of Duodenum and Jejunum

There were no effects of mycotoxins, YCWE, or interaction on villus height or villus width in duodenum of pigs (Table 5). Feeding diets with YCWE showed a tendency (*p* = 0.051) for reducing crypt depth in duodenum histomorphometry. No effects of mycotoxins, YCWE, or interaction were observed on villus height-to-crypt depth ratio in duodenum of pigs. Feeding diets with YCWE reduced (*p* < 0.05) villus width in jejunum. There were no effects of mycotoxins, YCWE, or interaction on villus height, crypt depth, and villus height-to-crypt depth ratio in jejunum of pigs.

### 2.2. Experiment 2

#### 2.2.1. Growth Performance

There were no effects of mycotoxins, YCWE, or interaction on body weight of pigs during the first 5 days among pigs in treatment groups (Table 6). Feeding diets with mycotoxins tended to reduce (*p* = 0.079) pig body weight at day 15 and significantly reduced (*p* < 0.05) pig body weight in all periods between days 20 and 48. Feeding diets with YCWE showed a tendency (*p* = 0.057) for reducing body weight of pig on days 10 and 20, and significantly decreased (*p* < 0.05) pig body weight on day 15. Feeding diets with mycotoxins tended to decrease (*p* = 0.020) ADG of pigs from day 10 to 15 and significantly reduced (*p* < 0.05) ADG from day 15 to 20, as well as for all periods between days 20 and 48. Feeding diets with YCWE decreased (*p* < 0.05) ADG from day 0 to 5, from day 5 to 10, and for the phase 1 period. There were no effects of mycotoxins, YCWE, or interaction on ADG of pigs from 20 to 27 days. There was a tendency (*p* = 0.096) for an interaction on ADG of pigs from 27 to 34 days, indicating that mycotoxins tended to reduce ADG but the tendency disappeared with YCWE addition. There was an interaction (*p* < 0.05) on pig ADG from day 34 to 41, indicating that mycotoxins reduced ADG but YCWE successfully recovered ADG reduction. Feeding diets with mycotoxins reduced (*p* < 0.05) ADG of pigs for all periods from day 27 until the end of the experimental period, phase 2, and the overall period. Feeding diets with YCWE reduced (*p* < 0.05) ADFI of pigs for the periods evaluated during the first 15 days of the study and tended to decrease (*p* = 0.065) ADFI during phase 1. Feeding diets with mycotoxins reduced (*p* < 0.05) ADFI of pigs for all periods from day 5 to the end of the study, for phase 1, phase 2, and the overall periods. Feeding diets with YCWE tended to lower (*p* = 0.054) G:F for the first 5 days of the study. There was a tendency (*p* = 0.054) for an interaction from day 5 to 10, where feeding diets with mycotoxins alone tended to increase G:F, whereas feeding diets with mycotoxins and YCWE tended to reduce G:F of pigs. There were no effects of mycotoxins, YCWE, or interaction for G:F from day 10 to 34 or for phase 1 of pigs. Feeding diets with mycotoxins tended to increase (*p* = 0.078) G:F of pigs from day 41 to 48, and significantly increased (*p* < 0.05) G:F during phase 2 and the overall periods. Feeding diets with YCWE increased (*p* < 0.05) G:F of pigs from day 35 to 48, as well as for phase 2, and tended to increase (*p* = 0.079) G:F in the overall period.

#### 2.2.2. Apparent Ileal Digestibility

There were no effects of mycotoxins, YCWE, or interaction for dry matter apparent ileal digestibility of pigs (Table 7). There was a tendency (*p* = 0.091) for an interaction, where feeding diets with mycotoxins alone reduced (*p* < 0.05) apparent ileal digestibility of crude protein, whereas feeding diets with mycotoxins and YCWE increased (*p* < 0.05) apparent ileal digestibility of crude protein of pigs. Similarly, there was a tendency (*p* = 0.096) for an interaction, where feeding diets with mycotoxins alone reduced (*p* < 0.05) apparent ileal digestibility of gross energy, whereas feeding diets with mycotoxins and YCWE increased (*p* < 0.05) apparent ileal digestibility of gross energy of pigs. There was an interaction (*p* < 0.05), where feeding diets with mycotoxins alone reduced (*p* < 0.05) apparent ileal digestibility of ether extract, whereas feeding diets with mycotoxins and YCWE increased (*p* < 0.05) apparent ileal digestibility of ether extract of pigs.

#### 2.2.3. Hematological Measurements

There were no effects of mycotoxins, YCWE, or interaction for hematological measurements on day 14 of pigs, except for neutrophils, where feeding diets with mycotoxins decreased (*p* < 0.05) the concentration in blood (Table 8). Feeding diets with YCWE increased (*p* < 0.05) red blood cell count, whereas it tended to increase (*p* = 0.071) white blood cell count at 45 days. Feeding diets with mycotoxins tended to increase (*p* = 0.052) hemoglobin concentration, whereas feeding diets with YCWE tended to reduce (*p* = 0.086) hemoglobin concentration on day 45. Feeding diets with YCWE reduced (*p* < 0.05) the percentage of hematocrit on day 45. There were no effects of mycotoxins, YCWE, or interaction for mean corpuscular volume, mean corpuscular hemoglobin, or mean corpuscular hemoglobin concentration of pigs on day 45. Feeding diets with YCWE tended to lower (*p* = 0.058) platelet count on day 45. Feeding diets with YCWE decreased (*p* < 0.05) lymphocyte count on day 45. There were no effects of mycotoxins, YCWE, or interaction for monocyte or eosinophil concentrations of pigs on day 45.

#### 2.2.4. Serum Biochemical Measurements

Feeding diets with mycotoxins tended to decrease (*p* = 0.077) total protein concentration in serum on day 45 (Table 9). Likewise, feeding diets with YCWE tended to reduce (*p* = 0.077) total protein concentration in serum at 45 days. Feeding diets with mycotoxins tended to increase (*p* = 0.078) albumin concentration on day 45 and to decrease (*p* = 0.071) globulin concentration on day 14. Feeding diets with mycotoxins increased (*p* < 0.05) the albumin-to-globulin ratio on day 14. There was an interaction (*p* < 0.05) for the albumin-to-globulin ratio on day 45, indicating that there was a reduction of the albumin-to-globulin ratio when YCWE was added for pigs fed diets with no mycotoxins. There was an interaction for cholesterol on day 45, indicating that there was a decrease in cholesterol level when pigs were fed diets with mycotoxins and YCWE, in comparison to when pigs were fed diets without mycotoxins but with YCWE. There was a tendency (*p* = 0.067) for an interaction for serum AST on day 14, indicating that YCWE tended to reduce serum AST in pigs fed diets with mycotoxins but the tendency disappeared in pigs fed diets without mycotoxins. Feeding diets with YCWE tended to decrease (*p* = 0.091) serum alkaline phosphatase on day 14. Feeding diets with mycotoxins increased (*p* < 0.05) creatinine concentration in serum on day 14. Feeding diets with mycotoxins reduced (*p* < 0.05) cholesterol in serum on day 14. Feeding diets with YCWE decreased (*p* < 0.05) calcium levels, tended to decrease (*p* = 0.083) sodium levels, and tended to increase (*p* = 0.081) chloride levels in serum on day 14. Feeding diets with mycotoxins tended to decrease (*p* = 0.064) potassium concentration and tended to increase (*p* = 0.053) sodium-to-potassium in serum on day 14. There were no effects of mycotoxins, YCWE, or interaction for alkaline phosphatase, BUN, BUN-to-creatinine ratio, phosphorus, glucose, and creatine phosphokinase in serum of pigs neither on day 14 nor 45.

#### 2.2.5. Jejunal Histomorphometry and Crypt Cell Proliferation

Feeding diets with mycotoxins reduced (*p* < 0.05) villus height in pig jejunum (Table 10). However, feeding diets with YCWE tended to increase (*p* = 0.088) villus height in pig jejunum. Feeding diets with mycotoxins tended to increase (*p* = 0.096) the width at the top of jejunal villus. There were no effects of mycotoxins, YCWE, or interaction for the width in the middle or bottom of the villus, nor for crypt depth and villus height/crypt depth ratio of pigs. Feeding diets with mycotoxins tended to reduce (*p* = 0.091) the percentage of Ki-67 staining-positive cells in jejunum. Whereas, feeding diets with YCWE tended to increase (*p* = 0.052) the percentage of cells positive to Ki-67 staining.

#### 2.2.6. Immunological and Oxidative Stress Measurements

Feeding diets with mycotoxins increased (*p* < 0.05) immunoglobulin A (IgA) concentration in jejunal mucosa of pigs. In the other hand, feeding diets with YCWE reduced (*p* < 0.05) IgA concentration in jejunal mucosa of pigs. Feeding diets with YCWE tended to reduce (*p* = 0.055) TNF-α concentration in jejunal mucosa of pigs. There was a tendency for an interaction (*p* = 0.083) for TNF-α concentration in pig serum on day 14, where feeding diets with mycotoxins alone increased (*p* < 0.05) TNF-α concentration in comparison to feeding diets with mycotoxins and YCWE (Table 11). There were no effects of mycotoxins, YCWE, or interaction for serum concentrations of TNF-α, IgA, protein carbonyl, or MDA on day 45 of pigs. On day 14, there were no effects of mycotoxins, YCWE, or interaction for serum concentrations of IgA, IgG, protein carbonyl, or MDA of pigs. There was a tendency (*p* = 0.057) for an interaction, where IgG concentration tended to be higher in pigs fed diets with only mycotoxins than in diets with mycotoxins and YCWE on day 45. There were no effects of mycotoxins, YCWE, or interaction for jejunal mucosa concentrations of IgG and MDA of pigs. There was an interaction (*p* < 0.05), where feeding mycotoxins alone increased protein carbonyl concentration in jejunal mucosa of pigs, whereas feeding mycotoxins and YCWE reduced the amount of protein carbonyl.

#### 2.2.7. Microbiome Analysis in Jejunal Mucosa

**Figure 1:**
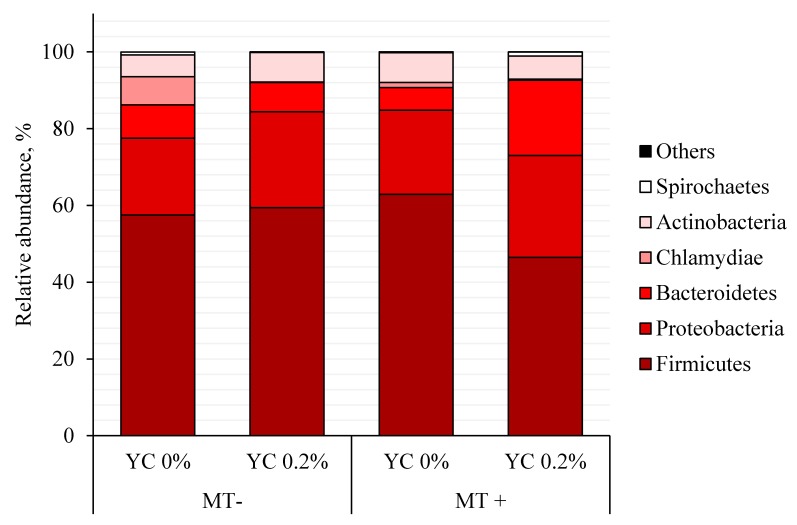
Bacterial phyla (expressed as a percentage of relative abundance of sequences) collected from jejunal mucosa of weanling pigs fed diets with mycotoxins (MT) or and yeast cell wall extract (YC), based on 16S rRNA gene sequencing in experiment 2. Each pattern represents a particular bacterial phylum. Phylum sequences that did not achieve 1% within each phylum were combined as “Others”. MT-: diet without aflatoxin B1 and fumonisin B1; MT+: inclusion of 180 μg/kg aflatoxin B1, 1 mg/kg deoxynivalenol, and 9 mg/kg fumonisin B1 by replacing the clean corn and clean wheat with naturally mycotoxin-contaminated corn and wheat; YC 0%: no addition of yeast cell wall extract (YCWE; Mycosorb A+, Alltech, Nicholasville, KY, USA); YC 0.2%: YCWE added at 2 g/kg of feed.

There were no effects of mycotoxins, YCWE, or interaction on pigs for the following bacterial phylum sequences from jejunal mucosa: Actinobacteria, Firmicutes, Proteobacteria, Chlamydiae, Deinococcus-Thermus, Fusobacteria, Nitrospirae, Tenericutes, and Verrucomicrobia (Table 12 and Figure 1). There was an interaction (*p* < 0.05) for Bacteriodetes indicating that YCWE increased Bacteriodetes in pigs fed diets with mycotoxins. There was a tendency (*p* = 0.062) for an interaction for Spirochaetes, indicating that YCWE tended to increase Spirochaetes in pigs fed diets with mycotoxins.

There were no effects of mycotoxins, YCWE, or interaction on pigs for the percentage of bacterial sequences from jejunal mucosa in pigs for the following bacterial families: Clostridiaceae, Veillonellaceae, Ruminococcaceae, Propionibacteriaceae, Helicobacteraceae, Moraxellaceae, Oxalobacteraceae, Oxalobacteraceae, Chlamydiaceae, Staphylococcaceae, Pseudomonadaceae, Streptococcaceae, Paenibacillaceae, Succinivibrionaceae, Xanthomonadaceae, and for the total percent of all families lower than 1.0% in each family (Table 13). Feeding diets with mycotoxins increased (*p* < 0.05) the percentage of sequences from the family Lactobacillaceae. Feeding diets with YCWE reduced (*p* < 0.05) the proportion of sequences from the family Prevotellaceae. Feeding diets with YCWE tended to decrease (*p* = 0.064) the proportion of sequences from *Eubacterium*. Feeding diets with YCWE decreased (*p* < 0.05) the percentage of sequences from Erysipelotrichaceae and tended to decrease (*p* = 0.064) the proportion for *Eubacterium* in that family.

There were no effects of mycotoxins, YCWE, or interaction on pigs for the percentage of bacterial sequences from jejunal mucosa for the following bacterial species: *Lactobacillus mucosae, Clostridium perfringens, Propionibacterium acnes, Lactobacillus delbrueckii, Chlamydia suis, Lactobacillus sp., Clostridium butyricum, Dialister succinatiphilus, Faecalibacterium prausnitzii, Succinivibrio dextrinosolvens, Massilia niabensis, Acinetobacter radioresistens, Streptococcus hyointestinalis, Mitsuokella jalaludinii, Ruminococcus gauvreauii, Helicobacter equorum, Staphylococcus sciuri, Stenotrophomonas rhizophila, Helicobacter mastomyrinus, Mitsuokella multacida, Prevotella sp., Helicobacter rappini, Bacillus coagulans, Eubacterium multiforme, Roseburia faecis, Clostridium hiranonis, Eubacterium biforme,* and *Lactobacillus johnsonii* (Table 14). Feeding diets with mycotoxins reduced (*p* < 0.05) the percentage of sequences from *Lactobacillus kitasatonis* in jejunal mucosa of pigs. Feeding diets with YCWE increased (*p* < 0.05) the proportion of sequences from *Prevotella copri* and *Prevotella stercorea,* whereas it decreased (*p* < 0.05) the proportion from *Lactobacillus equicursoris.* Feeding diets with YCWE tended to increase the proportion of sequences from *Turicibacter sanguinis* (*p* = 0.064) and *Clostridium sp.* (*p* = 0.075). Feeding diets with mycotoxins tended to decrease the proportion of sequences from *Leclercia adecarboxylata* (*p* = 0.064) and *Trabulsiella odontotermitis* (*p* = 0.067).

#### 2.2.8. Tight Junction Proteins in Jejunum

**Figure 2:**
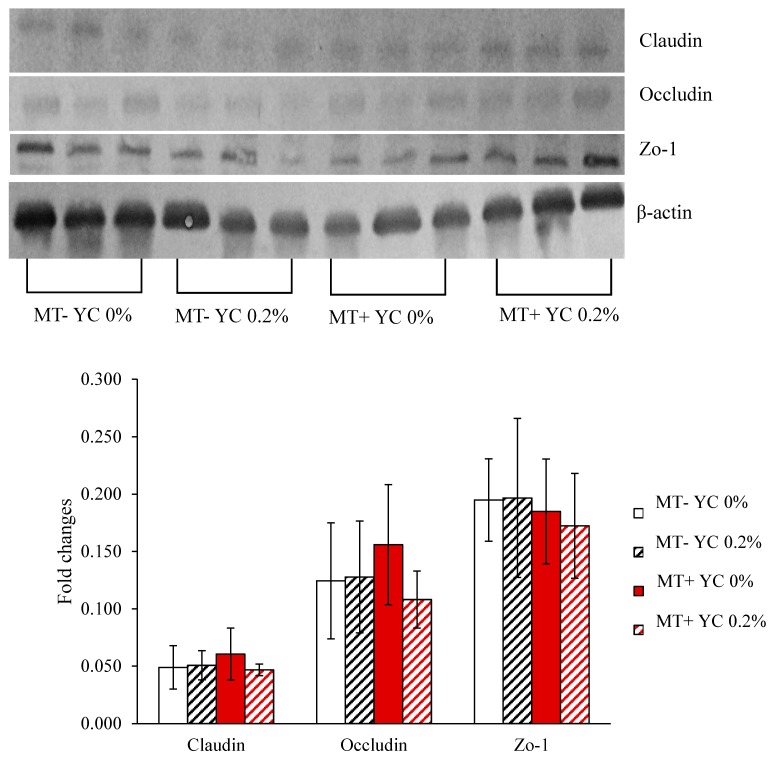
Tight junction proteins of jejunal mucosa in weanling pigs fed diets with mycotoxin or/and yeast cell wall extract. Zo-1: zona occludens-1 protein; MT-: diet without aflatoxin B1 and fumonisin B1; MT+: inclusion of 180 μg/kg aflatoxin B1, 1 mg/kg deoxynivalenol, and 9 mg/kg fumonisin B1 by replacing the clean corn and clean wheat with naturally mycotoxin-contaminated corn and wheat; YC 0%: no addition of yeast cell wall extract (YCWE; Mycosorb A+, Alltech, Nicholasville, KY, USA); YC 0.2%: YCWE added at 2 g/kg of feed.

There were no effects of mycotoxins, YCWE, or interaction for tight junction proteins in jejunal mucosa of pigs (Figure 2).

## 3. Discussion

The present study was designed to test the efficacy of the YCWE (Mycosorb A+, Alltech Inc. Kentucky) derived from the cell wall of *Saccharomyces cerevisiae* and algal material in nursery and growing pigs challenged with AFB1, DON, and FB1 in naturally contaminated diets (Table 15).

In nursery pigs, aflatoxin concentration up to 20 μg/kg has shown no impact on the growth of pigs [3]. Nevertheless, the aflatoxin concentration at 180 μg/kg used in experiment 2 has shown impairment on growth performance of nursery pigs, where a stronger impairment on growth performance was noticed in comparison to growing pigs from experiment 1. In experiment 2, feeding diets with mycotoxins had an obvious and negative effect on growth performance in nursery pigs by reducing BW, ADG, and ADFI by 11%, 13%, and 17%, respectively. However, mycotoxins increased G:F by 4% during the entire period. The compensatory improvement of G:F is possibly due to the reduction in feed intake [12]. Mycotoxin impact was weaker in growing pigs, where mycotoxins decreased ADG by 18% and G:F by 15% during only the first seven days. These results along with the absence of change in ADFI during the first seven days are indicative that mycotoxins impaired animal growth by affecting nutrient absorption or utilization during acute challenge. As a result, mycotoxins tended to reduce pig BW on day 14. Challenges with aflatoxin and fumonisin in growing pigs have been shown to reduce BW, ADG, and ADFI when in higher concentrations (2.5 mg of aflatoxin and 100 mg of FB1/kg) than the concentrations used in experiment 1 (180 μg/kg AFB1 and 14 mg/kg FB1) [13]. A compensatory improvement of G:F of 8% was observed in growing pigs from day 14 to 21 in experiment 1 from the current study. Of interest, the interaction observed from day 27 to 34 and from day 34 to 41 showed that the reduction on ADG in nursery pigs fed diets with mycotoxins was ceased by the inclusion of YCWE in diets, suggesting a protective role of YCWE against mycotoxins with an impact on pig growth performance. The effects of YCWE on growth performance of nursery pigs fed diets with mycotoxins is supported by the results obtained for nutrient digestibility, where the apparent ileal digestibility of crude protein, gross energy, and ether extract was reduced in pigs fed diets with mycotoxins but increased when YCWE was included in the diets.

Comparing the results of both experiments, along with results previously reported in the scientific field, the severity and persistence of the effects of the mycotoxin challenge on growth performance of pigs seems to depend on age, being more severe in nursery pigs in comparison to growing pigs. In addition, nursery pigs can be more susceptible to the mycotoxin challenge due to weaning stress [14]. Besides age and weaning stress factors, the presence of deoxynivalenol in the experimental diets fed to nursery pigs in experiment 2 may have intensified mycotoxins impairment on growth performance. Piglets weaned with 24 days (older than in experiment 2) and challenged with 3 mg/kg of DON, have shown impaired growth performance during the 14 days of the challenge [15]. However, deoxynivalenol-challenged pigs presented impaired growth performance when fed values as low as 0.6 mg/kg [16]. Deoxynivalenol impairment on growth performance seems to be caused specially by its anorexigenic effect. The reduction on feed intake caused by deoxynivalenol is due to an increased release of proinflammatory cytokines [17,18] satiety hormones [19], and neuroendocrine regulation [20]. Satiety hormones such as peptide YY and cholecystokinin have increased levels in serum after deoxynivalenol challenge in mice, resulting in reduced feed intake [19]. Resistance to deoxynivalenol was developed by adult mice where no anorexigenic effect was observed for at least two days after ceasing the challenge with the mycotoxin [21]. The same study showed that animals developed a dose-dependent increase in feed intake after the challenge, reinforcing animal ability of acclimation to mycotoxins. The neuroendocrine regulation is mediated by serotonin receptor activation in rats, reducing digesta transit time, and thus, feed intake [20]. Feeding higher concentrations of deoxynivalenol can even cause emesis. High serum levels of peptide YY and serotonin were described as responsible for the deoxynivalenol emetic effect in mice [22].

The YCWE from *S. cerevisiae* is composed of an inner layer of insoluble β-D-glucans arranged in a network [23]. Insoluble property and structural conformation allow the β-D-glucans to survive digestion and to mitigate the impact of mycotoxins in the lower gastrointestinal tract. Directly related to mycotoxins, β-D-glucans [8,24] and a mixture of YCWE, clay, and organic acids [15] have previously shown binding efficacy on *Fusarium* toxins, resulting in reduced deoxynivalenol impact on growth performance of nursery pigs [15]. Even though previous studies have shown improved animal performance, minor effects were observed in pigs fed diets with YCWE considering growth performance for nursery and growing pigs. In growing pigs, YCWE tended to reduce animal ADG during the first seven days and ADFI during the last seven days, suggesting a mild detrimental effect of YCWE on growth performance. In experiment 2 with nursey pigs, YCWE reduced feed intake during phase 1, which may have caused the reduced ADG for the same phase. The lower G:F for nursery pigs fed diets with YCWE during the first seven days may have enhanced G:F during the last days of the study, after day 34, as a compensatory mechanism [12], as observed as a tendency of increased G:F for the overall period for pigs fed diets with YCWE. The results regarding YCWE and growth performance were unexpected, since a combination of fermented media by *S. cerevisiae* and hydrolyzed yeast cell wall from *S. cerevisiae* [25] or *S. cerevisiae* cell wall [26] demonstrated enhanced nutrient digestibility and utilization in weaned pigs. Likewise, pigs fed YCWE had greater digestibility than pigs fed a basal diet without *S. cerevisiae* cell wall [26]. The production of total volatile fatty acids in ileum and cecum was also reported to be increased in pigs fed β-D-glucans from *S. cerevisiae* [27].

Growing pigs consuming mycotoxins showed increased serum albumin and reduced serum cholesterol and BUN. The observed increase in serum albumin could be due to the ability of the serum protein to hydrolyze *Fusarium* toxins [28] and to its antioxidant role [29], properties that may help with handling the mycotoxin challenge. Cholesterol and urea are primarily synthetized by hepatocytes and their decrease is indicative of impaired liver function [1]. In experiment 2, the interaction observed on day 45, where mycotoxins a decreased cholesterol level among pigs fed YCWE, is indicative that the supplementation with YCWE was not able to recover cholesterol synthesis by hepatocytes. Although, YCWE showed a tendency to reduce serum AST in pigs fed diets with mycotoxins. The aspartate aminotransferase is more sensitive than alanine aminotransferase for hepatic damage in pigs [30]. Therefore, YCWE could improve liver function in pigs with moderate hepatic damage, as shown in experiment 2 of the current study. The reduction on glucose and K levels, with a consequent impact on the Na-to-K ratio, might indicate kidney damage, due to inefficient glucose and K reabsorption after glomerular filtration [31,32,33]. The YCWE was able to prevent those changes in glucose and K. The tendency of increase in Na and Cl levels in serum of growing pigs fed diets with YCWE may be due to its composition. One of the components in the YCWE product is hydrated sodium calcium aluminosilicate, justifying the increase of Na in serum and the concomitant increase in Cl to maintain the electrolytic balance in serum.

The mycotoxin challenge can modulate the immune function of pigs and potentially increase animal susceptibility to infectious diseases or morbidity [34]. In the current study, mycotoxins reduced neutrophils blood levels for growing pigs in experiment 1. In growing pigs, the increased duodenal IgG concentration in animals fed mycotoxin compared to pigs without a mycotoxin challenge may indicate a late immune response, characterizing a specialized response by the adaptive immune system. In previous study carried out by our group, pigs challenged with DON and zearalenone did not show any alteration in white blood cells and IgG in comparison to animals fed a control diet [1]. In a different study, DON-challenged pigs have shown decreased serum IgG [33]. A combination of fermented media by *S. cerevisiae* and hydrolyzed yeast cell wall from *S. cerevisiae* have been shown to increase IgG and IgM levels in serum and IgA level in the gut [25]. In experiment 1, IgG levels in jejunum and serum, and lymphocyte count were increased by YCWE when pigs were fed diets without mycotoxins, suggesting YCWE’s ability to stimulate an immune response. In experiment 2, it was possible to notice a reduction of albumin-to-globulin ratio in pigs fed diets with YCWE and no mycotoxins, reinforcing YCWE’s effect as an immune stimulator. In experiment 1, equivalent IgG levels in jejunum were observed between pigs fed diets with YCWE and pigs fed diets with mycotoxins, indicating that YCWE could stimulate the immune system as much as mycotoxins but without the latter’s toxic effects. On the other hand, IgG concentration in serum in experiments 1 and 2 did not follow the same behavior as observed for the jejunal IgG described. Indeed, there was a reduction when YCWE was added to diets of pigs fed diets with mycotoxins, indicating a protective role of YCWE at the gut level with a possible reduction of systemic immune response.

The hypothesis of late response by the adaptive immune system is supported by no differences in TNF-α and MDA in gut mucosa and blood serum, which are related to early inflammatory response and cell damage, respectively. The absence of difference between pigs fed mycotoxin diets and control pigs for TNF-α and MDA were also observed in pigs challenged with *Fusarium* toxins [1]. A mixture of YCWE, clay, and organic acids was able to reduce TLR-4 expression and improve gut barrier function in deoxynivalenol-challenged nursery pigs [15]. Regarding cytokines, IFN-γ, IL-6, IL-12B, TNF-α, and PTGS2 were over expressed in pigs fed diets with DON [33]. The inclusion of YCWE in diets decreased 8-OHdG, molecule that indicates nucleic acid injury, suggesting reduced oxidative stress and improved cell viability [1]. Furthermore, the inclusion of YCWE in diets with mycotoxins successfully overcame the increase in protein carbonyl in jejunum mucosa observed in nursery pigs fed diets with mycotoxins.

Despite the effects observed for immune and oxidative stress markers, there were no noticeable effects on tight junction expression for claudin, occludin, or zona occludens-1 protein in experiment 2. Tight junctions are responsible for the juxtaposition of enterocytes and thus, are indicative of intestinal wall integrity [35]. The absence of effect of mycotoxins, YCWE, or interaction suggest that both the challenge with mycotoxins and the YCWE supplementation could not alter gut wall structure regarding tight junction constitution. Even still, there are other immune-related structures present on enterocyte surface that seem to have be altered in current study. The immune system can be modulated by the interaction of molecules present in the intestinal lumen with receptors along the intestinal wall. One such receptor is the TLR2, which can be stimulated by both yeast components as well as bacterial lipopolysaccharide [36,37]. The supplementation with YCWE could have enhanced the expression of TLR2 which promoted the survivability of Spirochaetes [36], as observed in experiment 2. The supplementation with the yeast cell wall has previously shown to reduce the proportion of disease-related bacteria [38]. In the current study, similar results were observed with the decrease of the proportion of Erysipellotrichaceae and Prevotellaceae families in pigs fed diets with YCWE, despite the increase in specific species within the family (*P. copri* and *P. stercorea*), suggesting an improvement in intestinal health of pigs fed diets with YCWE. This line of thought can also be used to explain the decrase in *Lactobacillus equicursoris* observed in pigs fed diets with YCWE. The *L. equicursoris* has demonstrated antagonistic ability against pathogenic bacteria in pigs [39], indicating that the supplementation with YCWE could have a role in reducing the load of pathogenic bacteria in the intestinal tract of pigs. The species *Trabulsiella odontotermitis* is found in the gastrointestinal tract of termites that are able to digest fungi cell wall [40]. The reduction of *T. odontotermitis* in pigs fed diets with mycotoxins was unexpected, since the ingestion of mycotoxins should have enhanced pigs’ ability to handle fungi presence in the feed. At the same time, pigs fed diets with mycotoxins had an increased proportion of Lactobalicaceae family and of *Lactobacillus kitasatonis*, which is known to play a probiotic role in pig intestine [41]. The *Lactobacillus* sp. have previously shown the ability to reduce mycotoxicity by binding to mycotoxins extracellularly [42,43] and, as gram-positive bacteria, can be considered as “native DON-degraders” [44]. Thus, the increase of Lactobacilaceae family proportion in the intestinal microbiome of mycotoxin-fed pigs as well as the increase of gram-positive bacteria as *Turicibacter sanguinis* and *Clostridium* sp. in pigs fed diets with YCWE can be indicative of an induced adaptation of the microbiome and of the pig itself to handle better mycotoxin challenge.

Cells from the intestinal crypts are responsible for enterocyte renewal and crypt depth is positively related with proliferative rate that can be measured by Ki-67 staining [45]. The tendency in reducing duodenal crypt depth in animals fed YCWE may indicate that the additive was able to enhance enterocyte survivability, thus reducing crypt cells’ proliferative rate. Indeed, YCWE tended to increase villus height as previously reported in pigs fed YCWE in comparison to pigs fed a basal diet without *S. cerevisiae* cell wall [26]. On the other hand, feeding diets with mycotoxins reduced villus height and tended to reduce the percentage of cells positive to Ki-67. Such outcomes suggest that mycotoxins reduced the villus height by impairing crypt cell proliferation. At the same time, the tendency to increase in villus width may be an adaptation strategy to increase the absorptive surface area after mycotoxins damage.

Collectively, the current study suggests that susceptibility of pigs to AFB1 180 μg/kg, DON 1 mg/kg, and FB1 9 mg/kg is higher in nursery pigs (6 to 29 kg, challenged for 48 days) than in growing pigs (56 to 89 kg, challenged for 35 days) to AFB1 180 μg/kg and FB1 14 mg/kg, depending on the health status of gastrointestinal and immune systems. The YCWE at 0.2% showed a protective role against the aforementioned mycotoxins, improving pig growth and health mainly in nursery pigs in comparison to growing pigs.

## 4. Materials and Methods

A protocol of these experiments was reviewed and approved by the Institutional Animal Care and Use Committee (IACUC) at North Carolina State University (NCSU; Raleigh, NC, USA).

### 4.1. Animals and Experimental Diets

In experiment 1, one hundred and twenty pigs (60 barrows and 60 gilts at 55.58 ± 3.13 kg, crossbred pigs, Smithfield Premium Genetics, Rose Hill, NC, USA) were used. Pigs were housed in solid concrete floor indoor pens (1.42 × 3.86 m) at the North Carolina State University Swine Evaluation Station (Clayton, NC, USA). Pigs were grouped by body weight (BW) and randomly assigned to four treatments within a BW group. Each treatment had ten replicates and three pigs per pen.

In experiment 2, forty-eight newly weaned pigs at 3 weeks of age (24 barrows and 24 gilts at 5.98 ± 0.24 kg, PIC 337 × Camborough 22) were used. This study was conducted at the North Carolina State University Metabolism Evaluation Unit (Raleigh, NC, USA). Pigs were housed in a pen (0.74 × 1.5 m) equipped with a polyethylene feeder attached to the front of the pen, nipple water next to the feeder, and slatted flooring. Pigs were grouped by body weight (BW) and randomly assigned to four treatments within a BW group. Each treatment had twelve replicates and one pig per pen.

Corn and wheat naturally contaminated with mycotoxins were identified and the mycotoxin concentrations were confirmed. Corn was analyzed by the Alltech 37+ program at Alltech (Nicholasville, KY, USA) for 16 mycotoxins including AFB1 and FB1, and wheat was analyzed for 11 mycotoxins including DON. Quantification of AFB1 and FB1 was conducted using UPLC-MS/MS. Corn contained AF (2.8 mg/kg for experiments 1 and 2) and FUM (170.2 mg/kg for experiments 1 and 2) and wheat contained DON (7.3 mg/kg, for experiment 2) and these were used to make experimental diets (Table 16 and Table 17). This contaminated corn and wheat were blended with corn and wheat without mycotoxins in order to reach the desired levels of 180 μg/kg AFB1 and 14 mg/kg FB1 (experiment 1) and 180 μg/kg AFB1, 1 mg/kg DON, and 9 mg/kg FB1 (experiment 2) in the final diets. Non-contaminated corn and wheat were also used to formulate a control without mycotoxins. Mycotoxin analysis in corn and wheat was completed by collecting 10 samples from different locations to obtain a representative mixture. Ten samples were combined and thoroughly blended together before two subsamples were collected for analysis of mycotoxin content measured by the Alltech 37+ program using UPLC-MS/MS (Table 18).

Four treatments were based on a 2 × 2 factorial arrangement with mycotoxin (180 μg/kg AFB1 and 14 mg/kg FB1 or 180 ug/kg AFB1, 1 mg/kg DON, and 9 mg/kg FB1 for experiments 1 or 2, respectively) and yeast cell wall extract (YCWE; Mycosorb A+, Alltech Inc., Nicholasville, KY, USA: 2 g/kg diet) as two factors. Thus, the 4 treatments were: (1) MT-YC 0%: corn-soybean meal-based diet without detectable AFB1 and FB1, (2) MT-YC 0.2%: MT-YC 0% + YCWE, (3) MT+YC 0%: MT-YC 0% + 180 μg/kg AFB1 and 14 mg/kg FB1 or 180 μg/kg AFB1, 1 mg/kg DON, and 9 mg/kg FB1 (respectively for experiments 1 or 2) by the use of naturally contaminated corn and wheat replacing clean corn and wheat used in MT-YC 0%, and (4) MT+YC 0.2%: MT+YC + YCWE. The YCWE-based MT-YC 0.2% and MT+YC 0.2% is composed of hydrolyzed yeast, which includes the cell wall fraction of the organism. Pigs were fed the experimental diets for a 5-week period based on the phase 5 diet for experiment 1, and were fed the experimental diets for 48 days based on a 2-phase feeding program (phase 1: 20 days and phase 2: 28 days). Feed intake and body weight were recorded weekly. During the entire experimental period, all pigs had free access to feed and water. Concentrations of essential nutrients met requirements suggested by the National Research Council [46]. All diets were free of antimicrobial growth promoter and ZnO. Titanium dioxide (0.4%) was added to experimental diets from day 43 of experiment 2 as an indigestible external marker to measure apparent ileal digestibility (AID).

Challenge periods and inclusion levels of mycotoxins in this study were based on previous studies where pigs at similar body weights were challenged with AF, FUM, and DON (isolated or in combination) for equivalent periods of time to enable comparison of results [2,4,16]. The inclusion level of 0.2% for YCWE was chosen based on the recommended level by the manufacturer, which was previously tested and showed the ability to reduce mycotoxin toxicity [3,47].

### 4.2. Sampling and Laboratory Analyses

#### 4.2.1. Blood Sampling

In experiment 1, the pig with median BW from each pen was bled at the end of 4 weeks of feeding, whereas in experiment 2, pigs were bled on days 14 and 45 for hematological, biochemical, anti-oxidative, and immunological analysis. Blood was collected in Vacutainer tubes (BD Biosciences, San Jose, CA, USA) without anticoagulant to obtain serum for serum biochemistry as indicators of overall physiological status, TNF-α as an indicator of inflammatory status, IgA (only for experiment 2) and IgG as indicators of humoral immune status, MDA as an indicator of lipid peroxidation, protein carbonyl (only for experiment 2) as an indicator of oxidative protein damage, and 8-hydroxydeoxyguanosine (8-OHdG, only for experiment 1) as an indicator of oxidative DNA damage. Blood was allowed to clot before centrifuging for 15 min at 3000 × *g* (4 °C) to collect serum, and samples were stored at –80 °C until analyzed. Blood samples were also collected in tubes containing EDTA to obtain whole blood for hematological measurements.

#### 4.2.2. Tissue, Mucosa, and Digesta Collection

At the last day of feeding, from each treatment, a pig representing an average body weight of each of the 8 pens (4 gilt pens and 4 barrow pens) were selected, excluding one of the heaviest and one of the lightest pens for experiment 1, and all pigs for experiment 2. Pigs were euthanized to collect mucosa tissue samples from duodenum (a portion of 20 cm, only for experiment 1) and distal jejunum (a portion of 20 cm prior to ileum). Duodenum and distal jejunum were isolated and flushed with saline solution. About 10 cm of their section was fixed in 10% formaldehyde phosphate buffer and kept for microscopic assessment of mucosal morphology, such as villus height and crypt depth. Mucosa from the left duodenum and jejunum were also stored in liquid nitrogen immediately after collection and moved to –80 °C until analysis. The mucosa was used to measure TNF-α, IgG, and MDA for both experiments and IgA and protein carbonyl only for experiment 2. In experiment 2, ileal portion (a portion of 20 cm prior to ileocecal valve) was used to obtain ileal digesta for apparent ileal digestibility. The ileal digesta was collected by gently squeezing. The ileal digesta were stored in sterile containers and kept frozen at –20 °C. The ileal digesta was freeze-dried (24D × 48, Virtis, Gardiner, NY) for storage and chemical analysis. Freeze-dried digesta was used for measuring apparent ileal digestibilityof dry matter, crude protein, growth energy, and ether extract. In experiment 2, jejunal tissues were used to measure tight junction proteins (claudin-1, occludin, and zona occludens-1 protein) as indicators of gut integrity using Western Blot.

#### 4.2.3. Hematological and Biochemical Assays

Whole blood with EDTA was sent to Antech Diagnostics (Cary, NC, USA) for complete blood counting. Measurements included hematocrit, hemoglobin, mean corpuscular hemoglobin, mean corpuscular hemoglobin concentration, mean corpuscular volume, platelet number, red blood cell count, white blood cell count, basophils, eosinophils, lymphocytes, monocytes, and neutrophils.

Concentrations of serum alanine aminotransferase, albumin, alkaline phosphatase, aspartate aminotransferase, bilirubin, BUN-to-creatinine ratio, calcium, chloride, cholesterol, creatinine, creatine phosphokinase, globulin, glucose, phosphorus, potassium, sodium, and BUN were measured (Antech Diagnostics, Cary, NC, USA) for determination of serum biochemistry.

#### 4.2.4. Tumor Necrosis Factor-α

Tumor necrosis factor-α was measured in duodenal and jejunal mucosa as well as in serum by enzyme-linked immunosorbent assay (ELISA), as described by Weaver et al. [48]. Determination of TNF-α was completed following the manufacturer’s procedure (PTA00; R and D System, Minneapolis, MN, USA). Mucosa samples (500 mg) of duodenum and jejunum were weighed and suspended into 1.0 mL PBS. Mucosa samples were homogenized (Tissuemiser; Thermo Fisher Scientific Inc., Rockford, IL, USA) on ice. The homogenate was centrifuged for 30 min at 10,000× *g* (4 °C) to collect supernatant, which was used to determine concentrations of TNF-α and protein concentrations. Protein concentrations of mucosa supernatant in duodenum and jejunum were measured using a BCA protein assay (23225; Thermo Fisher Scientific, Rockford, IL, USA). Briefly, 50 µL of standard plus dilute or 100 µL of sample was added to microplate wells coated with capture antibody in conjunction with biotinylated antibody reagent. Detection occurred by the use of horseradish peroxidase, TMB substrate, and a stop solution of 2 M sulfuric acid (H_2_SO_4_). Absorbance was read at 450 nm and 550 nm by an ELISA plate reader (Synergy HT, Biotek Inc. Winooski, VT, USA) and the Gen 5 data analysis software (Biotek Inc. Winooski, VT, USA). Concentrations of TNF-α in mucosa and serum were expressed as ng/g of protein and pg/mL, respectively. The detection limit for TNF-α was 5 pg/mL. Unless otherwise defined, all processes of sample extraction and protein measurements used herein have the same method.

#### 4.2.5. Immunoglobulins A and G

Concentrations of porcine IgA and IgG in duodenal and jejunal mucosa as well as in serum were measured via ELISA, as described by Weaver et al. [4]. Goat anti-pig IgA or IgG was used to capture antibodies by coating wells. Horseradish peroxidase conjugated to goat anti-pig IgA or IgG were used as the detection antibody in combination with the TMB (3,3′,5,5′-tetramethylbenzidene) enzyme substrate (E100-102 or E100-104; Bethyl Laboratories Inc., Montgomery, TX, USA). A solution of 0.18 M H_2_SO_4_ was used to stop the enzyme-substrate reaction. Absorbance was read at 450 nm using an ELISA plate reader and Gen 5 data analysis software. Concentrations of IgA and IgG in mucosa and serum were expressed as mg/g of protein and mg/mL, respectively. Detection limits were 15.6 to 1000 ng/mL or 7.8 to 500 ng/mL for IgA and IgG, respectively.

#### 4.2.6. Malondialdehydes

Concentrations of MDA in duodenal and jejunal mucosa as well as in serum were analyzed using a TBARS assay (STA-330; Cell Biolabs, San Diego, CA, USA) as described by Shen et al. [49]. Concentrations of MDA in mucosa and serum were expressed as µmol/mg protein and nmol/mL, respectively. The assay range for MDA was 0 to 125 µM.

#### 4.2.7. 8-Hydroxy-Deoxyguanosine

Production of 8-OHdG in serum was determined by ELISA (STA-320; Cell Biolabs, San Diego, CA, USA) as described by Weaver et al [48]. Undiluted samples were added to an 8-OHdG conjugate-coated microplate, followed by diluted anti-8-OHdG antibody, and finally diluted secondary antibody enzyme conjugate. After incubation, the provided stop solution was added to each well, and allowed to incubate for 8–10 min before being stopped with a stop solution in order to achieve a color change which was not over-saturated. Samples were then measured at 450 nm and concentration was determined based on the standard curve.

#### 4.2.8. Protein Carbonyl

Concentration of protein carbonyl in jejunal mucosa and serum as an index of oxidative protein was analyzed using an ELISA kit (STA 310; Cell Biolabs, San Diego, CA, USA) as described by Shen et al. [49]. Concentrations of protein carbonyl in mucosa and plasma were expressed as µmol/g protein. The assay range for protein carbonyl was 0 to 7.5 nmol/mg protein.

#### 4.2.9. Chemical Analysis

In experiment 2, dry matter of digesta was quantified by weighing digesta samples prior to and after freeze-drying, as described in Passos et al. [50]. Nitrogen was quantified in ground feed and digesta samples using TruSpec N Nitrogen Determinator (LECO Corp., St. Joseph, MI, USA) to calculate crude protein (method 992.15; [51]). Gross energy was quantified in ground feed and digesta samples using a Parr 6200 Calorimeter (Parr Instrument Co., Moline, IL). Ether extract was quantified in ground feed and digesta samples using ether extraction method (method 920.39; [51]).

#### 4.2.10. Apparent Ileal Digestibility

In experiment 2, apparent ileal digestibility (%) of dry matter, crude protein, gross energy, and ether extract was calculated using the titanium dioxide concentration in digesta and feed by using the equation: AID = 100 – ((ND/NF) × (TiF/TiD) × 100), where, AID is the apparent nutrient digestibility, ND is the nutrient concentration present in the ileal digesta, NF is the nutrient concentration in the feed, TiF is the titanium dioxide concentration in the feed, and TiD is the titanium dioxide concentration in the ileal digesta. Titanium dioxide was measured and calculated using a standard curve based on the methods described by Myers et al. [52].

#### 4.2.11. Immunohistochemistry for Ki-67 and Morphometry on Duodenum and Jejunum

In experiment 1, duodenal and jejunal samples were embedded in paraffin, cut cross-section to 5 µm thick, and mounted on polylysine-coated slides. Slides were then stained (hematoxylin and eosin) and examined under a Sony Van–Ox S microscope (Opelco, Washington, DC). Villus height (from the tip of the villi to the villus-crypt junction), villus width (width of the villus at one-half of the villus height), and crypt depth (from villus junction to the base of the crypt) were determined according to Weaver et al. [48]. Lengths of 10 well-oriented intact villi and their associated crypt were measured in each slide. The same person executed all the analyses of intestinal morphology.

In experiment 2, jejunal samples were embedded in paraffin. Epitope retrieval was performed using 10 mM citrate buffer, pH 6.0 in a pressure cooker (Dako, Carpinteria, CA). Endogenous peroxidase was quenched with 3% hydrogen peroxide and sections were blocked using protein block reagent (Dako, Carpinteria, CA). Primary monoclonal antibody of Ki-67 (Dako, Carpinteria, CA) was used after 1:500 dilutions. Secondary antibody was attached using Vector ImmPRESS anti-mouse polymer reagent (Vector Laboratories, Burlingame, CA) after 1:2 dilutions. Diaminobenzamine reagent (Vector Laboratories, Burlingame, CA) was used as the chromogen. Image JS software [53] was used for calculating the Ki-67-positive cell. Simultaneously, jejunal morphometry can be available with the immunohistochemistry.

#### 4.2.12. Microbiome Analysis of Jejunal Mucosa

In experiment 2, mucosa-associated microbiome was sequenced. DNA was extracted from jejual mucosa with QIAGEN’s QIAamp^®^ DNA Stool MiniKit (Qiagen, Crawley, UK). Samples were prepared for template preparation on the Ion Chef TM instrument and sequencing on the Ion S5 TM system (ThermoFisher Scientific, Inc., Wilmington, DE, USA). Variable regions V2, V3, V4, V6, V7, V8, and V9 of the 16S rRNA gene were amplified with the Ion 16S Metagenomics Kit (ThermoFisher Scientific, Inc., Wilmington, DE). Sequences (hypervariable regions) were processed using the Torrent Suite TM Software (version 5.2.2; ThermoFisher Scientific, Inc., Wilmington, DE) to produce “.bam” files for further analysis. Sequence data analysis, alignment to GreenGenes (anybody) and MicroSeq (experts) databases, alpha and beta diversity plot generation, and OTU table generation were performed by the Ion Reporter TM Software Suite of bioinformatics analysis tools (version 5.2.2; ThermoFisher Scientific, Inc., Wilmington, DE). Samples were analyzed using Ion Reporter’s Metagenomics 16S workflow powered by Qiime (version w1.1).

#### 4.2.13. Tight Junction Proteins in Jejunal Tissue

In experiment 2, four samples of jejunal tissue in each treatment were used to measure tight junction protein, as described by Yang et al. [37]. Tissue samples (50 mg) of jejunum were weighed and suspended into 0.5 mL RIPA lysis and extraction buffer containing 5 µL protease inhibitor cocktail. Tissue samples were homogenized on ice. The homogenate was centrifuged at 10,000 × *g* at 4 °C for 10 min to collect supernatant. Protein concentration of the supernatant was adjusted to 2 µg/µL by using a BCA protein assay, as mentioned above. The adjusted supernatant was denatured at 100 °C for 5 min in the water bath and was loaded in each well for SDS-PAGE. After SDS-PAGE, the gel was moved on polyvinylidene difluoride membrane for transferring a target protein to the membrane. Protein was electrophoretically transferred at 90 mV for 1 h. This was then blocked in 5% skim milk, and incubated (overnight at 4 °C) with primary antibodies against claudin, occluding, zona occludens-1 protein, and β-actin. The membrane was subsequently washed and incubated (1 h at room temperature) with horseradish-conjugated secondary antibodies. The immunoblot was developed with the DAB substrate kit (34002; Pierce, Rockford, IL). The density of bands was identified by using image analyzer software (LI-COR Biosciences, Lincoln, NE).

### 4.3. Data Analysis and Interpretation

In both experiment 1 and experiment 2, data from this study were analyzed based on a randomized complete block design by the Mixed model of SAS Software (Cary, NC, USA). The experimental unit was a pen. Factors were mycotoxin and YCWE. Factors, interaction between factors, and sex were the fixed effects and initial BW block was a random effect. Diets with or without mycotoxins were compared with the PDIFF option to evaluate if the mycotoxin effect was mitigated by YCWE once an interaction between mycotoxin and YCWE was found. Statistical differences among treatment means were considered significant with *p* < 0.05, whereas 0.05 ≤ *p* < 0.10 was used as the criteria for tendency.

## Figures and Tables

**Table 1:** Growth performance of pigs fed diets with mycotoxins (MT) and yeast cell wall extract (YCWE^1^) (YC) in experiment 1.

Mycotoxins (MT)	-		+			*p* Value		
YCWE (YC)	0%	0.2%	0%	0.2%	SEM	MT	YC	MT × YC
**Body Weight,** **kg**								
Initial	55.7	55.6	55.7	55.8	3.1	0.687	0.929	0.608
Day 7	59.7	58.8	58.8	58.6	3.6	0.151	0.176	0.405
Day 14	68.9	68.0	67.8	67.2	3.8	0.099	0.178	0.758
Day 21	75.8	74.7	74.7	74.8	4.1	0.444	0.409	0.335
Day 28	82.5	80.8	81.2	82.1	4.3	0.547	0.262	0.197
Day 35	90.5	88.3	88.5	88.4	4.5	0.255	0.205	0.228
**ADG^2^,** **kg**								
Day 0 to 7	1.342	1.078	1.047	0.937	0.211	0.048	0.088	0.470
Day 7 to 14	1.311	1.303	1.282	1.229	0.054	0.271	0.506	0.626
Day 14 to 21	0.985	0.956	0.987	1.083	0.118	0.221	0.520	0.231
Day 21 to 28	0.963	0.880	0.928	0.933	0.067	0.879	0.492	0.442
Day 28 to 35	1.142	1.074	1.037	1.012	0.105	0.220	0.484	0.745
Overall	1.124	1.055	1.057	1.052	0.071	0.201	0.178	0.249
**ADFI^3^,** **kg**								
Day 0 to 7	2.733	2.502	2.597	2.579	0.142	0.788	0.256	0.330
Day 7 to 14	3.075	2.825	2.828	2.847	0.170	0.174	0.166	0.107
Day 14 to 21	2.889	2.774	2.813	2.785	0.232	0.741	0.472	0.659
Day 21 to 28	2.588	2.606	2.430	2.683	0.233	0.781	0.358	0.421
Day 28 to 35	3.093	2.828	2.872	2.672	0.252	0.145	0.073	0.796
Overall	2.894	2.733	2.713	2.721	0.183	0.165	0.272	0.225
**G:F^4^**								
Day 0 to 7	0.472	0.423	0.396	0.364	0.064	0.044	0.216	0.798
Day 7 to 14	0.434	0.461	0.457	0.435	0.024	0.925	0.896	0.151
Day 14 to 21	0.340	0.346	0.353	0.390	0.022	0.098	0.209	0.372
Day 21 to 28	0.381	0.344	0.390	0.355	0.026	0.683	0.137	0.960
Day 28 to 35	0.369	0.382	0.358	0.379	0.019	0.697	0.339	0.788
Overall	0.389	0.388	0.390	0.387	0.007	0.937	0.627	0.865

^1^ YCWE: yeast cell wall extract; ^2^ ADG: average daily gain; ^3^ ADFI: average daily feed intake; ^4^ G:F: gain to feed ratio. MT-: diet without aflatoxin B1 and fumonisin B1; MT+: inclusion of 180 μg/kg aflatoxin B1 and 14 mg/kg fumonisin B1 by replacing the clean corn with naturally mycotoxin-contaminated corn; YC 0%: no addition of YCWE (Mycosorb A+, Alltech, Nicholasville, KY, USA); YC 0.2%: YCWE added at 2 g/kg of feed.

**Table 2:** Cell counts in blood of pigs fed diets with mycotoxins (MT) and YCWE^1^ (YC) on day 28 in experiment 1.

Mycotoxins (MT)	-		+			*p* Value		
YCWE (YC)	0%	0.2%	0%	0.2%	SEM	MT	YC	MT × YC
RBC^2^, 106/µL	6.84	6.90	7.05	6.80	0.23	0.687	0.601	0.387
Hemogblobin, g/dL	12.43	12.48	12.64	11.95	0.45	0.709	0.317	0.254
Hematocrit, %	40.91	40.63	41.05	38.79	1.38	0.495	0.189	0.317
MCV^3^, fL	59.70	58.70	58.39	57.22	1.19	0.124	0.286	0.988
MCH^4^, pg	18.22	18.12	17.96	17.62	0.36	0.217	0.527	0.755
MCHC^5^, g/dL	30.47	30.81	30.79	30.79	0.21	0.492	0.420	0.412
Platelet count, mL	185.9	144.0	133.7	152.4	27.1	0.394	0.618	0.237
WBC^6^, 10^3^/µL	20.52	21.43	19.91	18.83	1.36	0.236	0.950	0.470
Neutrophils, cell/mL	7.89	6.66	5.89	5.77	0.72	0.003	0.139	0.225
Lymphocytes, cell/mL	10.97^A^	13.05^B^	12.47^AB^	11.30^AB^	0.97	0.868	0.614	0.099
Monocytes, cell/mL	1.04	1.13	0.96	1.13	0.15	0.777	0.397	0.781
Eosinophils, cell/µL	543	499	518	484	84	0.812	0.636	0.949
Basophils, cell/µL	109	128	130	66	45	0.585	0.545	0.271

^1^ YCWE: yeast cell wall extract; ^2^ RBC: red blood cells; ^3^ MCV: mean corpuscular volume; ^4^ MCH: mean corpuscular hemoglobin; ^5^ MCHC: mean corpuscular hemoglobin concentration; ^6^ WBC: white blood cells. MT-: diet without aflatoxin B1 and fumonisin B1; MT+: inclusion of 180 μg/kg aflatoxin B1 and 14 mg/kg fumonisin B1 by replacing the clean corn with naturally mycotoxin-contaminated corn; YC 0%: no addition of YCWE (Mycosorb A+, Alltech, Nicholasville, KY, USA); YC 0.2%: YCWE added at 2 g/kg of feed. ^AB^ Means within a row lacking a common superscript tend to differ (0.05 ≤ *p* < 0.1).

**Table 3:** Hematological measurements in serum of pigs fed diets with mycotoxins (MT) and YCWE^1^ (YC) on day 28 in experiment 1.

Mycotoxins (MT)	-		+			*p* Value		
YCWE (YC)	0%	0.2%	0%	0.2%	SEM	MT	YC	MT × YC
Total protein, g/dL	6.44	6.18	6.49	6.36	0.12	0.309	0.090	0.573
Albumin, g/dL	3.60	3.48	3.62	3.73	0.08	0.042	0.950	0.074
Globulin, g/dL	2.84	2.70	2.87	2.66	0.11	0.948	0.106	0.731
A to G ratio^2^	1.28	1.29	1.27	1.40	0.06	0.389	0.231	0.319
AST^3^, U/L	23.25	27.88	22.72	20.36	2.71	0.111	0.712	0.144
ALT^4^, U/L	19.38	20.63	20.03	17.83	1.76	0.507	0.769	0.291
ALP^5^, U/L	132	143	149	157	10	0.098	0.301	0.947
CPK^6^, U/L	1,230	1,749	1,119	1,358	254	0.287	0.113	0.552
BUN^7^, mg/dL	14.75	13.75	13.08	12.33	1.84	0.088	0.336	0.868
Creatinine, mg/dL	1.30	1.40	1.30	1.25	0.08	0.197	0.629	0.189
BUN-to-creatinine ratio	11.50	10.25	10.07	10.34	1.85	0.385	0.526	0.330
Cholesterol, mg/dL	86.13	85.25	79.89	74.29	3.65	0.019	0.354	0.497
Glucose, mg/dL	72.50	77.13	82.90	79.27	4.14	0.051	0.873	0.189
Ca, mg/dL	10.59	10.54	10.66	10.76	0.13	0.232	0.821	0.519
Cl, mEq/L	100.6	101.5	100.0	101.1	0.86	0.384	0.081	0.807
Na, mEq/L	145.4	147.5	145.8	146.0	0.65	0.374	0.064	0.129
K, mEq/L	5.28	5.35	5.06	4.95	0.22	0.064	0.914	0.566
Na to K ratio	27.63	27.75	28.96	29.90	1.06	0.053	0.553	0.652
P, mg/dL	9.16	9.03	8.82	8.86	0.16	0.100	0.721	0.577

^1^ YCWE: yeast cell wall extract; ^2^ A to G ratio: albumin to globulin ratio; ^3^ AST: aspartate aminotransferase; ^4^ ALT: alanine aminotransferase; ^5^ ALP: alkaline phosphatase; ^6^ CPK: creatine phosphokinase; and ^7^ BUN: blood urea N. MT-: diet without aflatoxin B1 and fumonisin B1; MT+: inclusion of 180 μg/kg aflatoxin B1 and 14 mg/kg fumonisin B1 by replacing the clean corn with naturally mycotoxin-contaminated corn; YC 0%: no addition of YCWE (Mycosorb A+, Alltech, Nicholasville, KY, USA); YC 0.2%: YCWE added at 2 g/kg of feed.

**Table 4:** Immunological and oxidative stress parameters in duodenal and jejunal mucosa and serum of pigs fed diets with mycotoxins (MT) and YCWE^1^ (YC) in experiment 1.

Mycotoxins (MT)	-		+			*p* Value		
YCWE (YC)	0%	0.2%	0%	0.2%	SEM	MT	YC	MT × YC
**Immunological Parameters**								
Duodenal TNF-α^2^, pg/mg protein	8.79	6.96	8.69	8.91	0.81	0.261	0.331	0.217
Jejunal TNF-α, pg/mg protein	6.56	5.53	5.36	5.80	0.75	0.490	0.659	0.281
Serumal TNF-α, pg/mL	183	187	169	173	11	0.206	0.726	0.959
Duodenal IgG^3^, µg/mg protein	2.77	4.26	5.36	4.61	0.71	0.049	0.605	0.128
Jejunal IgG, µg/mg protein	1.75 ^a^	2.95 ^b^	2.96 ^b^	2.40 ^ab^	0.37	0.255	0.274	0.005
Serumal IgG, mg/mL	7.92	8.04	9.49	7.75	0.85	0.240	0.143	0.096
**Oxidative Stress Parameters**								
Duodenal MDA^4^, nmol/mg protein	0.82	0.73	0.68	0.96	0.12	0.701	0.415	0.123
Jejunal MDA, nmol/mg protein	1.14	1.20	0.99	1.08	0.38	0.561	0.747	0.959
Serumal MDA, µM	15.94	14.74	15.80	15.22	5.39	0.915	0.575	0.845
Serumal 8-OHdG^5^, ng/mL	1.29	0.93	1.66	0.26	0.49	0.737	0.049	0.235

^1^ YCWE: yeast cell wall extract; ^2^ TNF-α: tumor necrosis factor-alpha; ^3^ IgG: immunoglobulin G; ^4^ MDA: malondialdehydes; ^5^ 8-OHdG: 8-hydroxy-2’-deoxyguanosine. MT-: diet without aflatoxin B1 and fumonisin B1; MT+: inclusion of 180 μg/kg aflatoxin B1 and 14 mg/kg fumonisin B1 by replacing the clean corn with naturally mycotoxin-contaminated corn; YC 0%: no addition of YCWE (Mycosorb A+, Alltech, Nicholasville, KY, USA); YC 0.2%: YCWE added at 2 g/kg of feed. ^ab^ Means within a row lacking a common superscript differ (*p* < 0.05).

**Table 5:** Intestinal morphology of pigs fed diets with mycotoxins (MT) and YCWE^1^ (YC) in experiment 1.

Mycotoxins (MT)	-		+			*p* Value		
YCWE (YC)	0%	0.2%	0%	0.2%	SEM	MT	YC	MT × YC
**Duodenum**								
Villus height (VH), µm	516	484	498	491	19	0.770	0.306	0.509
Villus width, µm	115	112	111	108	5.8	0.323	0.442	1.000
Crypt depth (CD), µm	295	253	275	268	13	0.840	0.051	0.141
VH to CD ratio	1.77	1.93	1.83	1.83	0.08	0.761	0.192	0.233
**Jejunum**								
Villus height, µm	482	495	495	495	36	0.845	0.845	0.842
Villus width, µm	119	101	116	108	5.3	0.685	0.018	0.324
Crypt depth, µm	238	235	243	245	8.9	0.368	0.950	0.798
VH-to-CD ratio	2.04	2.11	2.05	2.01	0.16	0.758	0.899	0.646

^1^ YCWE: yeast cell wall extract. MT-: diet without aflatoxin B1 and fumonisin B1; MT+: inclusion of 180 μg/kg aflatoxin B1 and 14 mg/kg fumonisin B1 by replacing the clean corn with naturally mycotoxin-contaminated corn; YC 0%: no addition of YCWE (Mycosorb A+, Alltech, Nicholasville, KY, USA); YC 0.2%: YCWE added at 2 g/kg of feed.

**Table 6:** Growth performance of weanling pigs fed diets with mycotoxins (MT) and YCWE^1^ (YC) in experiment 2.

Mycotoxins (MT)	-		+			*p* Value		
YCWE (YC)	0%	0.2%	0%	0.2%	SEM	MT	YC	MT × YC
**Body Weight,** **kg**								
Initial	6.0	6.0	6.0	6.0	0.2	1.000	0.632	0.905
Day 5	6.1	5.9	5.9	5.9	0.3	0.364	0.258	0.569
Day 10	6.9	6.7	6.8	6.4	0.3	0.293	0.057	0.473
Day 15	8.5	8.1	8.2	7.6	0.4	0.079	0.037	0.779
Day 20	10.8	10.3	10.2	9.3	0.4	0.030	0.066	0.638
Day 27	14.5	14.3	13.7	14.4	0.6	0.017	0.156	0.327
Day 34	19.6	18.6	17.4	16.4	0.7	0.001	0.129	0.940
Day 41	25.4	24.3	22.2	22.0	0.8	<0.001	0.370	0.514
Day 48	31.7	30.4	27.6	27.9	1.0	<0.001	0.589	0.348
**ADG^2^, kg**								
Day 0 to 5	0.017	−0.017	−0.008	−0.024	0.014	0.205	0.048	0.489
Day 5 to 10	0.165	0.155	0.180	0.104	0.021	0.390	0.047	0.122
Day 10 to 15	0.321	0.278	0.265	0.243	0.029	0.082	0.210	0.673
Day 15 to 20	0.462	0.445	0.403	0.343	0.033	0.020	0.249	0.527
Day 20 to 27	0.529	0.567	0.506	0.444	0.044	0.103	0.783	0.259
Day 27 to 34	0.728 ^A^	0.614 ^AB^	0.521 ^B^	0.569 ^AB^	0.047	0.011	0.488	0.096
Day 34 to 41	0.831 ^a^	0.819 ^a^	0.686 ^b^	0.795 ^a^	0.034	0.006	0.110	0.045
Day 41 to 48	0.895	0.876	0.778	0.852	0.036	0.046	0.420	0.180
Phase 1 (day 0 to 20)	0.241	0.215	0.210	0.166	0.017	0.025	0.048	0.612
Phase 2 (day 20 to 48)	0.746	0.719	0.623	0.665	0.024	0.001	0.750	0.151
Overall	0.494	0.467	0.416	0.416	0.017	<0.001	0.420	0.440
**ADFI^3^, kg**								
Day 0 to 5	0.083 ^c^	0.053 ^d^	0.066	0.049	0.008	0.212	0.005	0.403
Day 5 to 10	0.234	0.201	0.201	0.145	0.019	0.027	0.027	0.564
Day 10 to 15	0.433	0.402	0.372	0.303	0.024	0.002	0.039	0.428
Day 15 to 20	0.642	0.625	0.523	0.488	0.039	0.001	0.475	0.805
Day 20 to 27	0.786	0.805	0.643	0.630	0.043	<0.001	0.944	0.722
Day 27 to 34	1.060	0.944	0.783	0.789	0.056	<0.001	0.328	0.281
Day 34 to 41	1.245	1.136	1.036	1.046	0.064	0.010	0.327	0.284
Day 41 to 48	1.529	1.406	1.275	1.262	0.055	<0.001	0.156	0.245
Phase 1 (day 0 to 20)	0.348	0.320	0.291	0.247	0.019	0.001	0.065	0.679
Phase 2 (day 20 to 48)	1.155	1.073	0.934	0.932	0.042	<0.001	0.301	0.324
Overall	0.752	0.700	0.613	0.589	0.028	<0.001	0.149	0.554
**G:F^4^**								
Day 0 to 5	−0.043	−1.346	−0.299	−1.109	0.542	0.986	0.054	0.646
Day 5 to 10	0.718	0.769	0.873 ^A^	0.632 ^B^	0.074	0.904	0.210	0.055
Day 10 to 15	0.732	0.693	0.721	0.806	0.061	0.352	0.678	0.256
Day 15 to 20	0.731	0.705	0.771	0.709	0.036	0.547	0.226	0.632
Day 20 to 27	0.666	0.695	0.791	0.703	0.045	0.140	0.501	0.190
Day 27 to 34	0.689	0.640	0.663	0.716	0.034	0.447	0.949	0.117
Day 34 to 41	0.675	0.731	0.663	0.769	0.022	0.547	<0.001	0.263
Day 41 to 48	0.587	0.626	0.604	0.678	0.019	0.078	0.006	0.366
Phase 1 (day 0 to 20)	0.693	0.666	0.716	0.673	0.032	0.579	0.186	0.766
Phase 2 (day 20 to 48)	0.649	0.673	0.666	0.717	0.012	0.016	0.003	0.287
Overall	0.660	0.673	0.679	0.708	0.012	0.023	0.079	0.495

^1^ YCWE: yeast cell wall extract; ^2^ ADG: average daily gain; ^3^ ADFI: average daily feed intake; ^4^ G:F: gain to feed ration. MT-: diet without aflatoxin B1 and fumonisin B1; MT+: inclusion of 180 μg/kg aflatoxin B1, 1 mg/kg deoxynivalenol, and 9 mg/kg fumonisin B1 by replacing the clean corn and clean wheat with naturally mycotoxin-contaminated corn and wheat; YC 0%: no addition of YCWE (Mycosorb A+, Alltech, Nicholasville, KY, USA); YC 0.2%: YCWE added at 2 g/kg of feed. ^ab^ Means within a row lacking a common superscript differ (*p* < 0.05). ^AB^ Means within a row lacking a common superscript tend to differ (0.05 ≤ *p* < 0.1).

**Table 7:** Apparent ileal digestibility (AID) in weanling pigs fed diets with mycotoxins (MT) and YCWE^1^ (YC) in experiment 2.

Mycotoxins (MT)	-		+			*p* Value		
YCWE (YC)	0%	0.2%	0%	0.2%	SEM	MT	YC	MT × YC
**AID, %**								
Dry matter	69.7	70.0	64.0	70.7	2.39	0.243	0.106	0.144
Crude protein	78.5	78.9	75.5 ^b^	80.1 ^a^	1.25	0.479	0.049	0.091
Gross energy	70.9 ^AB^	70.9 ^AB^	65.1 ^B^	72.6 ^A^	2.35	0.354	0.097	0.096
Ether extract	81.8 ^ab^	80.8 ^ab^	76.6 ^b^	83.5 ^a^	1.88	0.483	0.113	0.032

^1^ YCWE: yeast cell wall extract. MT-: diet without aflatoxin B1 and fumonisin B1; MT+: inclusion of 180 μg/kg aflatoxin B1, 1 mg/kg deoxynivalenol, and 9 mg/kg fumonisin B1 by replacing the clean corn and clean wheat with naturally mycotoxin-contaminated corn and wheat; YC 0%: no addition of YCWE (Mycosorb A+, Alltech, Nicholasville, KY, USA); YC 0.2%: YCWE added at 2 g/kg of feed. ^ab^ Means within a row lacking a common superscript differs (*p* < 0.05). ^AB^ Means within a row lacking a common superscript tend to differ (0.05 ≤ *p* < 0.1).

**Table 8:** Hematology of weanling pigs fed diets containing mycotoxins (MT) and YCWE^1^ (YC) on days 14 and 45 in experiment 2.

Mycotoxins (MT)	-		+			*p* Value		
YCWE (YC)	0%	0.2%	0%	0.2%	SEM	MT	YC	MT × YC
**RBC^2^, 10^6^/µL**								
Day 14	6.84	6.90	7.05	6.80	0.23	0.687	0.601	0.387
Day 45	6.86	6.30	6.91	6.68	0.17	0.194	0.023	0.313
**Hemoglobin, g/dL**								
Day 14	12.43	12.48	12.64	11.95	0.45	0.709	0.317	0.254
Day 45	12.11	11.51	12.53	12.18	0.27	0.052	0.086	0.659
**Hematocrit, %**								
Day 14	40.91	40.63	41.05	38.79	1.38	0.495	0.189	0.317
Day 45	41.58	38.92	42.33	40.17	1.15	0.392	0.043	0.830
**MCV^3^, fL**								
Day 14	59.70	58.70	58.39	57.22	1.19	0.124	0.286	0.988
Day 45	60.50	61.75	61.25	60.25	0.85	0.660	0.883	0.192
**MCH^4^, pg**								
Day 14	18.22	18.12	17.96	17.62	0.36	0.217	0.527	0.755
Day 45	17.67	18.31	18.14	18.32	0.25	0.330	0.104	0.347
**MCHC^5^, g/dL**								
Day 14	30.47	30.81	30.79	30.79	0.21	0.492	0.420	0.412
Day 45	29.21	29.70	29.66	30.40	0.41	0.170	0.147	0.757
**Platelet count, cell/mL**								
Day 14	185.9	144.0	133.7	152.4	27.1	0.394	0.618	0.237
Day 45	230.4	174.0	220.3	154.1	31.4	0.635	0.058	0.876
**WBC^6^, 10^3^/µL**								
Day 14	20.52	21.43	19.91	18.83	1.36	0.236	0.950	0.470
Day 45	18.62	16.30	18.68	16.25	1.28	0.997	0.071	0.966
**Neutrophils, cell/mL**								
Day 14	7.89	6.66	5.89	5.77	0.72	0.003	0.139	0.225
Day 45	3.64	4.21	3.89	3.45	0.44	0.545	0.882	0.233
**Lymphocytes, cell/mL**								
Day 14	10.97	13.05	12.47	11.30	0.97	0.868	0.614	0.100
Day 45	13.33	9.96	12.96	11.37	1.19	0.662	0.042	0.452
**Monocytes, cell/mL**								
Day 14	1.04	1.13	0.96	1.13	0.15	0.777	0.397	0.781
Day 45	1.07	1.04	1.32	0.98	0.18	0.580	0.285	0.377
**Eosinophils, cell/µL**								
Day 14	543	499	518	484	84	0.812	0.636	0.949
Day 45	502	451	478	434	78	0.786	0.529	0.954

^1^ YCWE: yeast cell wall extract; ^2^ RBC: red blood cells; ^3^ MCV: mean corpuscular volume; ^4^ MCH: mean corpuscular hemoglobin; ^5^ MCHC: mean corpuscular hemoglobin concentration; ^6^ WBC: white blood cells. MT-: diet without aflatoxin B1 and fumonisin B1; MT+: inclusion of 180 μg/kg aflatoxin B1, 1 mg/kg deoxynivalenol, and 9 mg/kg fumonisin B1 by replacing the clean corn and clean wheat with naturally mycotoxin-contaminated corn and wheat; YC 0%: no addition of YCWE (Mycosorb A+, Alltech, Nicholasville, KY, USA); YC 0.2%: YCWE added at 2 g/kg of feed.

**Table 9:** Biochemical blood assay of weanling pigs fed diets containing mycotoxins (MT) or and YCWE^1^ (YC) on days 14 and 45 in experiment 2.

Mycotoxins (MT)	-		+			*p* Value		
YCWE (YC)	0%	0.2%	0%	0.2%	SEM	MT	YC	MT × YC
**Total protein, g/dL**								
Day 14	4.65	4.64	4.67	4.57	0.09	0.742	0.542	0.606
Day 45	5.18	5.14	5.14	4.87	0.110	0.077	0.077	0.163
**Albumin, g/dL**								
Day 14	2.65	2.58	2.70	2.69	0.06	0.164	0.506	0.605
Day 45	3.33	2.98	7.64	5.27	1.96	0.078	0.457	0.583
**Globulin, g/dL**								
Day 14	2.00	2.06	1.97	1.82	0.08	0.071	0.539	0.167
Day 45	1.84	3.50	3.88	1.78	1.16	0.895	0.850	0.114
**Albumin to globulin ratio**								
Day 14	1.34	1.27	1.42	1.51	0.06	0.012	0.891	0.175
Day 45	1.84 ^a^	1.47 ^b^	1.62 ^ab^	1.76 ^ab^	0.09	0.723	0.218	0.009
**AST^2^, U/L**								
Day 14	33.92 ^AB^	38.83 ^AB^	41.42 ^A^	32.42 ^B^	3.69	0.884	0.583	0.067
Day 45	21.33	23.42	31.08	23.50	4.60	0.193	0.463	0.201
**ALT^3^, U/L**								
Day 14	22.17	25.50	22.42	24.33	1.85	0.806	0.165	0.704
Day 45	22.08	21.50	22.17	28.75	2.30	0.120	0.201	0.128
**ALP^4^, U/L**								
Day 14	381	352	375	332	21	0.535	0.091	0.718
Day 45	311	256	271	278	22	0.693	0.278	0.165
**CPK^5^, U/L**								
Day 14	1230	1749	1119	1358	254	0.287	0.113	0.552
Day 45	1224	1161	1931	1454	403	0.223	0.508	0.610
**BUN^6^, mg/dL**								
Day 14	15.42	17.75	16.50	17.67	1.61	0.751	0.270	0.711
Day 45	10.83	11.50	10.33	11.00	0.52	0.345	0.210	1.000
**Creatinine, mg/dL**								
Day 14	0.75	0.75	0.78	0.88	0.04	0.035	0.154	0.154
Day 45	0.88	0.89	0.89	0.82	0.04	0.351	0.351	0.245
**BUN-to-creatinine ratio**								
Day 14	20.42	24.08	21.33	20.42	2.09	0.455	0.455	0.216
Day 45	12.67	13.25	11.75	13.58	0.791	0.715	0.135	0.435
**Cholesterol, mg/dL**								
Day 14	86.13	85.25	79.89	74.29	3.65	0.019	0.354	0.497
Day 45	82.83 ^ab^	86.25 ^a^	83.92 ^ab^	77.08 ^b^	2.19	0.072	0.439	0.024
**Glucose, mg/dL**								
Day 14	101.3	104.3	99.1	99.4	4.0	0.377	0.680	0.741
Day 45	110.8	113.1	112.9	113.0	3.9	0.757	0.719	0.738
**Ca, mg/dL**								
Day 14	11.64	11.12	11.30	10.79	0.25	0.153	0.030	0.971
Day 45	11.31	10.96	11.02	11.13	0.14	0.683	0.416	0.108
**Cl, mEq/L**								
Day 14	100.6	101.5	100.0	101.1	0.86	0.384	0.081	0.807
Day 45	101.6	102.8	101.3	102.1	0.615	0.442	0.112	0.726
**Na, mEq/L**								
Day 14	144.4	143.3	145.3	143.5	0.86	0.534	0.083	0.678
Day 45	141.9	142.8	140.5	142.3	0.790	0.217	0.099	0.552
**K, mEq/L**								
Day 14	5.28	5.35	5.06	4.95	0.22	0.064	0.914	0.566
Day 45	5.57	6.08	5.68	5.53	0.25	0.381	0.479	0.181
**Na-to-K ratio**								
Day 14	27.63	27.75	28.96	29.90	1.06	0.053	0.553	0.652
Day 45	26.00	24.00	25.42	26.42	1.06	0.363	0.618	0.140
**P, mg/dL**								
Day 14	9.20	9.06	9.03	8.52	0.27	0.184	0.227	0.493
Day 45	10.76	10.91	10.55	10.54	0.24	0.246	0.773	0.748

^1^ YCWE: yeast cell wall extract; ^2^ AST: aspartate aminotransferase; ^3^ ALT: alanine aminotransferase; ^4^ ALP: alkaline phosphatase; ^5^ BUN: blood urea N; and ^6^ CPK: creatine phosphokinase. MT-: diet without aflatoxin B1 and fumonisin B1; MT+: inclusion of 180 μg/kg aflatoxin B1, 1 mg/kg deoxynivalenol, and 9 mg/kg fumonisin B1 by replacing the clean corn and clean wheat with naturally mycotoxin-contaminated corn and wheat; YC 0%: no addition of YCWE (Mycosorb A+, Alltech, Nicholasville, KY, USA); YC 0.2%: YCWE added at 2 g/kg of feed. ^ab^ Means within a row lacking a common superscript differ (*p* < 0.05). ^AB^ Means within a row lacking a common superscript tend to differ (0.05 ≤ *p* < 0.1).

**Table 10:** Jejunal morphology and crypt cell proliferation of weanling pigs fed diets with mycotoxins (MT) or and YCWE^1^ (YC) in experiment 2.

Mycotoxins (MT)	-		+			*p* Value		
YCWE (YC)	0%	0.2%	0%	0.2%	SEM	MT	YC	MT × YC
Villus height (VH), μm	521	525	509	520	4.4	0.047	0.088	0.448
Villus width (top), μm	91	89	91	100	3.1	0.096	0.246	0.130
Villus width (middle), μm	118	113	111	114	3.3	0.401	0.673	0.258
Villus width (bottom), µm	124	119	121	117	4.3	0.476	0.257	0.870
Crypt depth (CD), μm	239	239	232	234	4.3	0.101	0.764	0.820
VH-to-CD ratio^2^	2.19	2.20	2.20	2.23	0.04	0.759	0.632	0.788
Ki-67, %	26.0	28.7	22.0	26.5	2.01	0.091	0.052	0.646

^1^ YCWE: yeast cell wall extract; ^2^ Ki-67: technique for staining proliferating cells in the crypt where results are showed as a percentage of proliferating cells in comparison to all cells in the crypt. MT-: diet without aflatoxin B1 and fumonisin B1; MT+: inclusion of 180 μg/kg aflatoxin B1, 1 mg/kg deoxynivalenol, and 9 mg/kg fumonisin B1 by replacing the clean corn and clean wheat with naturally mycotoxin-contaminated corn and wheat; YC 0%: no addition of YCWE (Mycosorb A+, Alltech, Nicholasville, KY, USA); YC 0.2%: YCWE added at 2 g/kg of feed.

**Table 11:** Immune response and oxidative stress markers of serum and jejunum in weanling pigs fed diets with mycotoxins (MT) or and YCWE^1^ (YC) in experiment 2.

Mycotoxins (MT)	-		+			*p* Value		
YCWE (YC)	0%	0.2%	0%	0.2%	SEM	MT	YC	MT × YC
Tumor necrosis factor-α								
Serum at day 14, pg/mL	124 ^A^	124 ^A^	151 ^B^	127 ^A^	6.8	0.031	0.084	0.083
Serum at day 45, pg/mL	61	61	68	61	2.5	0.180	0.115	0.147
Jejunal mucosa, ng/mg protein	708	668	730	562	58	0.429	0.055	0.233
Immunoglobulin A								
Serum at day 14, mg/mL	0.42	0.33	0.41	0.37	0.05	0.701	0.183	0.554
Serum at day 45, mg/mL	0.57	0.55	0.67	0.61	0.06	0.187	0.515	0.752
Jejunal mucosa, μg/mg protein	5.83	5.57	6.85	6.12	0.24	0.002	0.037	0.318
Immunoglobulin G								
Serum at day 14, mg/mL	3.19	3.59	3.33	3.05	0.30	0.469	0.831	0.226
Serum at day 45, mg/mL	2.62 ^AB^	2.83 ^AB^	3.04 ^A^	2.51 ^B^	0.21	0.787	0.418	0.057
Jejunal mucosa, μg/mg protein	1.04	1.42	1.11	1.02	0.19	0.379	0.441	0.208
Protein carbonyl								
Serum at day 14, nmol/mg protein	1.91	2.00	2.16	1.85	0.15	0.730	0.458	0.189
Serum at day 45, nmol/mg protein	1.76	2.01	2.02	1.87	0.12	0.605	0.668	0.108
Jejunal mucosa, nmol/mg protein	2.33	2.31	2.83 ^a^	2.51 ^b^	0.08	0.001	0.026	0.047
Malondialdehydes								
Serum at day 14, μM	8.90	9.49	11.17	9.91	1.23	0.255	0.774	0.429
Serum at day 45, μM	8.89	9.57	10.25	9.83	1.43	0.542	0.922	0.682
Jejunal mucosa, nmol/g protein	511	542	488	579	78	0.925	0.401	0.679

^1^ YCWE: yeast cell wall extract. MT-: diet without aflatoxin B1 and fumonisin B1; MT+: inclusion of 180 μg/kg aflatoxin B1, 1 mg/kg deoxynivalenol, and 9 mg/kg fumonisin B1 by replacing the clean corn and clean wheat with naturally mycotoxin-contaminated corn and wheat; YC 0%: no addition of YCWE (Mycosorb A+, Alltech, Nicholasville, KY, USA); YC 0.2%: YCWE added at 2 g/kg of feed. ^ab^ Means within a row lacking a common superscript differ (*p* < 0.05). ^AB^ Means within a row lacking a common superscript tend to differ (0.05 ≤ *p* < 0.1).

**Table 12:** Bacterial phyla (expressed as a percentage of sequences) collected from jejunal mucosa of weanling pigs fed diets with mycotoxins (MT) or and YCWE^1^ (YC), based on 16S rRNA gene sequencing in experiment 2.

Mycotoxins (MT)	-		+			*p* Value		
YCWE (YC)	0%	0.2%	0%	0.2%	SEM	MT	YC	MT × YC
Actinobacteria	5.65	7.69	7.71	6.01	1.90	0.915	0.922	0.288
Bacteroidetes	8.64 ^ab^	7.66 ^ab^	5.93 ^b^	19.63 ^a^	3.52	0.182	0.069	0.037
Firmicutes	57.49	59.40	62.92	46.44	6.68	0.514	0.210	0.116
Proteobacteria	20.06	25.01	21.88	26.57	4.90	0.733	0.331	0.979
Spirochaetes	0.74 ^AB^	0.16 ^AB^	0.10 ^A^	1.07 ^B^	0.45	0.737	0.632	0.062
Chlamydiae	7.37	0.08	1.34	0.25	2.80	0.302	0.142	0.275
Deinococcus-Thermus	0.00	0.00	0.11	0.00	0.06	0.324	0.324	0.324
Fusobacteria	0.02	0.00	0.00	0.00	0.01	0.324	0.324	0.324
Nitrospirae	0.01	0.00	0.00	0.03	0.01	0.467	0.467	0.231
Tenericutes	0.01	0.00	0.00	0.01	0.01	0.965	0.965	0.161
Verrucomicrobia	0.00	0.00	0.01	0.00	0.01	0.324	0.324	0.324

^1^ YCWE: yeast cell wall extract. MT-: diet without aflatoxin B1 and fumonisin B1; MT+: inclusion of 180 μg/kg aflatoxin B1, 1 mg/kg deoxynivalenol, and 9 mg/kg fumonisin B1 by replacing the clean corn and clean wheat with naturally mycotoxin-contaminated corn and wheat; YC 0%: no addition of YCWE (Mycosorb A+, Alltech, Nicholasville, KY, USA); YC 0.2%: YCWE added at 2 g/kg of feed. ^ab^ Means within a row lacking a common superscript differ (*p* < 0.05). ^AB^ Means within a row lacking a common superscript tend to differ (0.05 ≤ *p* < 0.1).

**Table 13:** Bacterial families and genera (expressed as a percentage of sequences) collected from jejunal mucosa of weanling pigs fed diets with mycotoxins (MT) or and YCWE^1^ (YC), based on 16S rRNA gene sequencing in experiment 2.

Mycotoxins (MT)	-		+			*p* Value		
YCWE (YC)	0%	0.2%	0%	0.2%	SEM	MT	YC	MT × YC
Lactobacillaceae	13.95	20.02	32.20	35.86	6.68	0.011	0.451	0.851
*Lactobacillus*	13.95	20.02	32.20	35.86	6.68	0.011	0.451	0.851
Clostridiaceae	12.94	7.53	7.81	11.60	3.99	0.895	0.841	0.256
*Clostridium*	12.94	7.53	7.81	11.60	3.99	0.895	0.841	0.256
Prevotellaceae	14.84	7.77	11.38	3.73	3.78	0.295	0.044	0.935
*Prevotella*	14.84	7.77	11.38	3.73	3.78	0.295	0.044	0.935
Veillonellaceae	3.81	4.32	4.18	4.98	1.66	0.758	0.696	0.929
*Dialister*	0.10	0.31	0.22	0.13	0.10	0.762	0.576	0.129
*Mitsuokella*	0.52	1.56	1.75	2.75	0.91	0.192	0.271	0.981
Ruminococcaceae	2.37	3.54	2.65	4.57	2.03	0.707	0.379	0.831
*Faecalibacterium*	0.52	2.23	1.03	3.29	1.68	0.581	0.169	0.849
*Ruminococcus*	1.85	1.31	1.62	1.28	0.62	0.830	0.471	0.870
Propionibacteriaceae	2.46	5.03	2.59	2.86	0.93	0.249	0.110	0.194
*Propionibacterium*	0.02	0.00	0.01	0.00	0.01	0.542	0.215	0.542
Helicobacteraceae	2.50	5.10	0.80	3.92	2.90	0.623	0.329	0.930
*Helicobacter*	2.50	5.10	0.80	3.92	2.90	0.623	0.329	0.930
Bacillaceae	2.33	4.13	2.97	2.76	1.78	0.835	0.658	0.575
*Anoxybacillus*	0.50	1.63	1.32	0.12	0.69	0.615	0.957	0.097
*Bacillus*	1.65	2.35	1.55	2.46	1.66	0.999	0.631	0.949
Moraxellaceae	1.89	2.01	2.57	4.44	1.16	0.188	0.397	0.453
*Acinetobacter*	1.89	2.01	2.57	4.44	1.16	0.188	0.397	0.453
Lachnospiraceae	2.78	2.28	3.37	1.97	0.89	0.871	0.267	0.601
*Roseburia*	1.31	1.24	0.88	0.99	0.54	0.533	0.973	0.868
Oxalobacteraceae	1.06	0.25	0.16	0.34	0.34	0.244	0.365	0.160
*Massilia*	0.00	0.00	0.01	0.00	0.00	0.324	0.324	0.324
Enterobacteriaceae	3.65	0.83	1.42	2.90	1.29	0.919	0.632	0.097
*Leclercia*	0.12	0.00	0.00	0.00	0.06	0.324	0.324	0.324
*Proteus*	0.38	0.00	0.01	0.2	0.15	0.233	0.217	0.187
*Trabulsiella*	0.03	0.00	0.00	0.00	0.01	0.175	0.175	0.175
*Turicibacter*	0.34	0.19	1.35	0.38	0.52	0.125	0.144	0.241
Chlamydiaceae	0.04	8.24	0.70	0.05	2.79	0.185	0.184	0.121
*Chlamydia*	0.04	8.24	0.70	0.05	2.79	0.185	0.184	0.121
Staphylococcaceae	1.17	5.08	0.81	1.91	2.13	0.413	0.247	0.513
*Staphylococcus*	0.01	0.10	0.02	0.05	0.06	0.742	0.319	0.627
Pseudomonadaceae	0.68	3.19	1.61	1.63	1.04	0.752	0.212	0.216
*Pseudomonas*	0.58	2.96	1.52	1.43	1.03	0.760	0.247	0.212
Erysipelotrichaceae	2.72	0.45	2.93	0.98	0.92	0.665	0.017	0.853
Streptococcaceae	1.72	0.88	2.85	1.07	1.02	0.481	0.166	0.615
*Streptococcus*	1.72	0.88	2.85	1.07	1.02	0.481	0.166	0.615
Paenibacillaceae	6.17	0.75	0.17	0.15	2.08	0.121	0.198	0.201
Succinivibrionaceae	1.65	2.04	1.00	1.16	0.62	0.206	0.642	0.841
*Succinivibrio*	1.65	2.04	1.00	1.16	0.62	0.206	0.642	0.841
Xanthomonadaceae	3.88	0.20	0.35	0.53	1.79	0.369	0.326	0.279
*Stenotrophomonas*	0.04	0.00	0.00	0.03	0.02	0.673	0.673	0.121
Others^2^	17.44	16.36	17.60	12.62	3.74	0.634	0.423	0.604

^1^ YCWE: yeast cell wall extract; ^2^ Total percent combined of all family lower than 1.0% in each family. MT-: diet without aflatoxin B1 and fumonisin B1; MT+: inclusion of 180 μg/kg aflatoxin B1, 1 mg/kg deoxynivalenol, and 9 mg/kg fumonisin B1 by replacing the clean corn and clean wheat with naturally mycotoxin-contaminated corn and wheat; YC 0%: no addition of YCWE (Mycosorb A+, Alltech, Nicholasville, KY, USA); YC 0.2%: YCWE added at 2 g/kg of feed.

**Table 14:** Bacterial species (expressed as a percentage of sequences) collected from jejunal mucosa of weanling pigs fed diets with mycotoxins (MT) or and YCWE^1^ (YC), based on 16S rRNA gene sequencing in experiment 2^2^.

Mycotoxins (MT)	-		+			*p* Value		
YCWE (YC)	0%	0.2%	0%	0.2%	SEM	MT	YC	MT × YC
*Lactobacillus mucosae*	8.18	16.63	7.17	9.19	3.83	0.259	0.164	0.389
*Prevotella copri*	2.49	8.85	5.62	11.62	3.02	0.306	0.036	0.951
*Lactobacillus kitasatonis*	17.58	5.50	3.01	1.06	4.51	0.031	0.107	0.241
*Clostridium perfringens*	7.07	3.18	5.34	8.56	3.82	0.636	0.930	0.358
*Propionibacterium acnes*	2.85	2.55	5.00	2.43	0.92	0.245	0.104	0.197
*Lactobacillus delbrueckii*	2.35	2.64	2.57	1.59	0.50	0.386	0.476	0.190
*Chlamydia suis*	0.05	0.70	8.22	0.04	2.78	0.184	0.184	0.120
*Lactobacillus sp.*	2.04	2.38	2.01	1.19	0.49	0.159	0.582	0.179
*Lactobacillus equicursoris*	2.99	0.37	3.90	0.13	1.49	0.820	0.039	0.701
*Clostridium butyricum*	2.15	2.77	1.00	1.05	0.93	0.133	0.725	0.759
*Dialister succinatiphilus*	2.75	1.75	1.56	0.52	0.91	0.192	0.271	0.981
*Faecalibacterium prausnitzii*	1.28	1.62	1.31	1.85	0.62	0.830	0.471	0.870
*Succinivibrio dextrinosolvens*	1.16	1.00	2.04	1.65	0.62	0.206	0.642	0.841
*Massilia niabensis*	1.51	1.60	1.49	1.23	0.56	0.679	0.858	0.715
*Acinetobacter radioresistens*	2.43	1.49	0.94	0.72	0.75	0.139	0.441	0.631
*Prevotella stercorea*	0.70	1.95	0.89	2.03	0.58	0.813	0.047	0.921
*Streptococcus hyointestinalis*	0.90	2.04	0.51	1.52	0.96	0.597	0.217	0.938
*Mitsuokella jalaludinii*	0.95	1.02	1.25	1.57	0.52	0.414	0.713	0.808
*Ruminococcus gauvreauii*	2.22	0.29	1.88	0.38	1.42	0.917	0.161	0.859
*Helicobacter equorum*	0.08	0.00	2.94	1.65	1.66	0.182	0.681	0.718
*Staphylococcus sciuri*	0.00	0.67	3.52	0.22	1.79	0.396	0.465	0.273
*Turicibacter sanguinis*	0.59	1.18	0.26	2.37	0.71	0.544	0.064	0.286
*Stenotrophomonas rhizophila*	0.24	0.24	0.10	3.73	1.77	0.349	0.311	0.312
*Helicobacter mastomyrinus*	2.22	0.41	1.25	0.41	1.04	0.642	0.207	0.643
*Mitsuokella multacida*	0.99	0.83	0.96	1.28	0.55	0.696	0.875	0.659
*Leclercia adecarboxylata*	1.65	0.73	0.48	0.64	0.40	0.064	0.257	0.106
*Prevotella sp.*	0.49	0.54	1.17	1.17	0.54	0.160	0.965	0.958
*Helicobacter rappini*	1.61	0.39	0.90	0.39	0.82	0.665	0.296	0.659
*Bacillus coagulans*	1.35	0.81	0.18	0.51	0.68	0.285	0.875	0.529
*Clostridium sp.*	0.21	0.41	0.19	1.74	0.48	0.182	0.075	0.164
*Eubacterium multiforme*	0.40	0.22	0.79	1.10	0.45	0.170	0.891	0.593
*Roseburia faecis*	0.46	0.70	0.65	0.68	0.34	0.806	0.684	0.749
*Trabulsiella odontotermitis*	1.11	0.53	0.33	0.40	0.28	0.067	0.292	0.190
*Clostridium hiranonis*	0.18	0.13	0.85	1.11	0.56	0.118	0.839	0.771
*Eubacterium biforme*	0.34	1.37	0.18	0.30	0.40	0.124	0.148	0.255
*Anoxybacillus kestanbolensis*	0.06	0.97	1.06	0.08	0.48	0.913	0.945	0.056
*Lactobacillus johnsonii*	0.52	0.96	0.38	0.17	0.32	0.324	0.324	0.324

^1^ YCWE: yeast cell wall extract; ^2^ Species that are lower than 0.5% in each species had their values combined in the common genera. MT-: diet without aflatoxin B1 and fumonisin B1; MT+: inclusion of 180 μg/kg aflatoxin B1, 1 mg/kg deoxynivalenol, and 9 mg/kg fumonisin B1 by replacing the clean corn and clean wheat with naturally mycotoxin-contaminated corn and wheat; YC 0%: no addition of YCWE (Mycosorb A+, Alltech, Nicholasville, KY, USA); YC 0.2%: YCWE added at 2 g/kg of feed.

**Table 15:** Experimental design and mycotoxin contamination in feedstuff and diets for experiments 1 and 2.

Experiment	1				2			
Treatments	MT- YC 0%	MT- YC 0.2%	MT+ YC 0%	MT+ YC 0.2%	MT- YC 0%	MT- YC 0.2%	MT+ YC 0%	MT+ YC 0.2%
**Factor**								
Mycotoxin (MT)	-	-	+	+	-	-	+	+
YCWE^1^ (YC)	-	+	-	+	-	+	-	+
**Pigs**								
Per treatment	30	30	30	30	12	12	12	12
Per pen	3	3	3	3	1	1	1	1
**Period, d**	**35**	**35**	**35**	**35**	**48**	**48**	**48**	**48**
**Feedstuff**								
Ground yellow corn								
Aflatoxins, mg/kg	ND	ND	2.8	2.8	ND	ND	2.8	2.8
Fumonisins, mg/kg	ND	ND	170.2	170.2	ND	ND	170.2	170.2
Zearalenone, mg/kg	ND	ND	1.1	1.1	ND	ND	1.1	1.1
Wheat, soft red								
Deoxynivalenol, mg/kg	-	-	-	-	ND	ND	7.3	7.3
Zearalenone, mg/kg	-	-	-	-	ND	ND	1.8	1.8
**Diet^2^**								
YCWE, %	-	0.2	-	0.2	-	0.2	-	0.2
Aflatoxin B1, μg/kg	-	-	180	180	-	-	180	180
Fumonisin B1, mg/kg	-	-	14	14	-	-	9	9
Deoxynivalenol, mg/kg	-	-	-	-	-	-	1	1

^1^ YCWE: yeast cell wall extract; ^2^ Contaminated corn and wheat were blended with corn and wheat without mycotoxins in order to reach desired levels of mycotoxins in diets. Mycotoxin levels in feedstuff were detected by UPLC-MS/MS using Alltech 37+ program at Alltech (Nicholasville, KY, USA). ND: Not detected.

**Table 16:** Composition of experimental diets in experiment 1 (%, as-fed basis)^1^.

Item	Basal Diet
Ingredients, %	
Ground yellow corn	75.60
Soybean meal, dehulled	21.00
L-Lys HCl	0.18
Poultry fat	1.00
Salt	0.22
Vitamin premix^2^	0.03
Trace mineral premix^3^	0.15
Dicalcium P	1.12
Ground limestone	0.70
Calculated composition	
Dry matter, %	89.06
Metabolizable energy, Mcal/kg	3.35
Crude protein, %	16.49
Standardized ileal digestible Lys, %	0.85
Ca, %	0.60
Available P, %	0.27

^1^ Basal diet (MT-YC 0%) without aflatoxin B1 and fumonisin B1; MT-YC 0.2%: MT-YC 0% + 2 g/kg of a yeast cell wall extract (Mycosorb A+, Alltech, Nicholasville, KY, USA); MT+YC 0%: 180 μg/kg aflatoxin B1 and 14 mg/kg fumonisin B1 by the use of naturally contaminated corn replacing clean corn used in MT-YC 0%; and MT+YC 0.2%: MT+YC 0% + 2 g/kg of a yeast cell wall extract; Naturally contaminated corn contained aflatoxins (2.8 mg/kg) and fumonisins (170.2 mg/kg); ^2^ The vitamin premix provided the following per kilogram of complete diet: 6613.8 IU of vitamin A as vitamin A acetate; 992.07 IU of vitamin D3; 19.84 IU of vitamin E; 0.026 mg of vitamin B12; 4.63 mg of riboflavin; 26.46 mg of niacin; 18.52 mg of d-pnatothenic acid; 2.65 mg of Vitamin K as menadione sodium bisulfate; 0.66 mg of biotin; ^3^ The trace mineral premix provided the following per kilogram of complete diet: 39.6 mg of Mn as manganous oxide; 165 mg of Fe as ferrous sulfate; 165 mg of Zn as Zinc sulfate; 15.15 mg of Cu as copper sulfate; 0.30 mg of I as ethyenediamine dihydroiodide; and 0.30 mg of Se as sodium selenite.

**Table 17:** Composition of experimental diets in experiment 2 (%, as-fed basis)^1^.

Item	Phase 1	Phase 2
Ingredient, %		
Ground yellow corn	37.5	48.82
Soybean meal, dehulled	22.0	27.0
Wheat, soft red	15.0	15.0
Whey permeate	12.0	2.0
Poultry meal	5.0	3.0
Blood plasma	3.3	--
L-Lys HCl	0.45	0.41
DL-Met	0.17	0.12
L-Thr	0.13	0.12
Salt	0.22	0.22
Vitamin and mineral premix^2^	0.18	0.18
Dicalcium P	0.45	0.79
Ground limestone	1.10	0.84
Poultry fat	2.5	1.50
Calculated composition:		
Metabolizable energy, Mcal/kg	3.4	3.4
Crude protein, %	22.09	21.12
Standardized ileal digestible Lys, %	1.35	1.23
Standardized ileal digestible Met + Cys	0.75	0.68
Standardized ileal digestible Thr, %	0.79	0.73
Standardized ileal digestible Trp, %	0.23	0.22
Standardized total tract digestible P, %	0.40	0.33

^1^ Basal diet (MT-YC 0%) without aflatoxin B1 and fumonisin B1; MT-YC 0.2%: MT-YC 0% + 2 g/kg of a yeast cell wall extract (Mycosorb A+, Alltech, Nicholasville, KY, USA); MT+YC 0%: 180 μg/kg aflatoxin B1, 1 mg/kg DON, and 9 mg/kg fumonisin B1 by the use of naturally contaminated corn and wheat replacing clean corn and wheat used in MT-YC 0%; and MT+YC 0.2%: MT+YC 0% + 2 g/kg of a yeast cell wall extract; Naturally contaminated corn contained aflatoxins (2.8 mg/kg) and fumonisins (170.2 mg/kg) and naturally contaminated wheat contained deoxynivalenol (7.3 mg/kg); ^2^ The vitamin premix provided the following per kilogram of complete diet: 6613.8 IU of vitamin A as vitamin A acetate; 992.07 IU of vitamin D3; 19.84 IU of vitamin E; 0.026 mg of vitamin B12; 4.63 mg of riboflavin; 26.46 mg of niacin; 18.52 mg of d-pnatothenic acid; 2.65 mg of Vitamin K as menadione sodium bisulfate; 0.66 mg of biotin; ^2^ The trace mineral premix provided the following per kilogram of complete diet: 39.6 mg of Mn as manganous oxide; 165 mg of Fe as ferrous sulfate; 165 mg of Zn as Zinc sulfate; 15.15 mg of Cu as copper sulfate; 0.30 mg of I as ethyenediamine dihydroiodide; and 0.30 mg of Se as sodium selenite.

**Table 18:** Mycotoxin levels in corn and wheat used in Experiments 1 and 2 analyzed by UPLC-MS/MS^1^.

Mycotoxin	Corn	Wheat
Aflatoxin B1, mg/kg	2.5	ND
Aflatoxin B2, mg/kg	0.1	ND
Aflatoxin G1, mg/kg	0.2	ND
Deoxynivalenol (DON), mg/kg	ND	5.5
DON-3-glucoside, mg/kg	ND	0.9
15-acetyl-DON, mg/kg	ND	0.5
3-acetyl- DON, mg/kg	ND	0.3
Fumonisin B1, mg/kg	142.9	0.1
Fumonisin B2, mg/kg	13.9	ND
Fumonisin B3, mg/kg	13.4	ND
Fusarenon X, mg/kg	ND	ND
Gliotoxin, mg/kg	ND	ND
Neosolaniol, mg/kg	ND	ND
Nivalenol, mg/kg	0.2	ND
Ochratoxin A, mg/kg	0.1	ND
Ochratoxin B, mg/kg	ND	ND
Zearalenone, mg/kg	1.1	1.8

^1^ Mycotoxin analysis was performed by UPLC-MS/MS using the Alltech 37+ program at Alltech (Nicholasville, KY, USA).
